# Exposure to obesogenic endocrine disrupting chemicals and obesity among youth of Latino or Hispanic origin in the United States and Latin America: A lifecourse perspective

**DOI:** 10.1111/obr.13245

**Published:** 2021-05-05

**Authors:** Wei Perng, Alejandra Cantoral, Diana C. Soria‐Contreras, Larissa Betanzos‐Robledo, Katarzyna Kordas, Yun Liu, Ana M. Mora, Camila Corvalan, Anita Pereira, Marly Augusto Cardoso, Jorge E. Chavarro, Carrie V. Breton, John D. Meeker, Kim G. Harley, Brenda Eskenazi, Karen E. Peterson, Martha Maria Tellez‐Rojo

**Affiliations:** ^1^ Department of Epidemiology, Colorado School of Public Health University of Colorado Denver Anschutz Medical Campus Aurora Colorado USA; ^2^ Lifecourse Epidemiology of Adiposity and Diabetes (LEAD) Center University of Colorado Denver Anschutz Medical Campus Aurora Colorado USA; ^3^ National Council of Science and Technology National Institute of Public Health Mexico City Mexico; ^4^ Center for Nutrition and Health Research National Institute of Public Health Mexico City Mexico; ^5^ Department of Epidemiology and Environmental Health School of Public Health and Health Professions Buffalo New York USA; ^6^ Department of Epidemiology Brown University Providence Rhode Island USA; ^7^ Center for Environmental Research and Children's Health, School of Public Health University of California Berkeley Berkeley California USA; ^8^ Central American Institute for Studies on Toxic Substances (IRET) Universidad Nacional de Costa Rica Heredia Costa Rica; ^9^ Institute of Nutrition and Food Technology University of Chile Santiago Chile; ^10^ Department of Nutrition, School of Public Health University of São Paulo São Paulo Brazil; ^11^ Department of Nutrition and Epidemiology Harvard T.H. Chan School of Public Health Boston Massachusetts USA; ^12^ Division of Environmental Health University of Southern California Keck School of Medicine Los Angeles California USA; ^13^ Department of Environmental Health Sciences University of Michigan School of Public Health Ann Arbor Michigan USA; ^14^ Department of Nutritional Sciences University of Michigan School of Public Health Ann Arbor Michigan USA

**Keywords:** child health, lifecourse epidemiology, obesity, obesogenic endocrine disrupting chemicals

## Abstract

Following a 2019 workshop led by the Center for Global Health Studies at the Fogarty International Center on the topic of childhood obesity prevention and research synergies transpiring from cross‐border collaborations, we convened a group of experts in the United States and Latin America to conduct a narrative review of the epidemiological literature on the role of obesogenic endocrine disrupting chemicals (EDCs) in the etiology of childhood obesity among Latino youth in the United States and Latin America. In addition to summarizing and synthesizing results from research on this topic published within the last decade, we place the findings within a lifecourse biobehavioral framework to aid in identification of unique exposure‐outcome relationships driven by both biological and behavioral research, identify inconsistencies and deficiencies in current literature, and discuss the role of policy regulations, all with the goal of identifying viable avenues for prevention of early life obesity in Latino/Hispanic populations.

**Abbreviations**
BBzP: butylbenzyl phthalate
BMI: body mass index
BPA: bisphenol A
CCCEH: Columbia Center for Children's Environmental Health
CHAMACOS: Center for the Health Assessment of Mothers and Children of Salinas
DAP: dialkyl phosphate
DBP: dibutyl phthalate
DDE: dichlorodiphenyldichloroethylene
DDT: dichlorodiphenyltrichloroethane
DEHP: di(2‐ethylhexyl) phthalate
DEP: diethyl phthalate
DiBP: diisobutyl phthalate
EDCs: endocrine disrupting chemicals
ELEMENT: Early Life Exposure in Mexico to Environmental Toxicants
MBP: mono‐*n*‐butyl phthalate
MBzP: monobenzyl phthalate
MCPP: mono‐(3‐carboxylpropyl) phthalate
MEHP: mono(2‐ethylhexyl) phthalate
MEP: monoethyl phthalate
MiBP: mono‐isobutyl phthalate
NHANES: National Health and Nutrition Examination Survey
OPs: organophosphorous pesticides
PAHO: Pan‐American Health Organization
PBDEs: polybrominated diphenyl ethers
PFAS: perfluoroalkyl substances
PFHxS: perfluorohexane sulfonate
PFNA: perfluorononanoate
PFOA: perfluorooctanoate
PFOS: perfluorooctane sulfonate
PON1: paraoxonase 1
POPs: persistent organic pollutants

## 1 INTRODUCTION

The World Health Organization estimates that obesity worldwide has tripled since the 1970s,^1^ with 62% of adults categorized as overweight or obese in the Americas. The high prevalence of obesity has spared no age group, including young children.^2^, ^3^ Obesity during early life is concerning due to the short‐term metabolic and psychosocial consequences of excess adiposity, as well as the lifelong implications for cardiometabolic disease risk.^4^, ^5^, ^6^

In the United States, children and adolescents of Latino/Hispanic descent have approximately twofold higher prevalence of obesity than their White counterparts.^7^ While the root causes of this discrepancy are multifaceted, socioeconomic and ethnic inequities are key drivers. In Latin America, many countries are undergoing the epidemiological transition, related to a process of modernization and urbanization that coincides with increased life expectancy.^8^ The industrial advancements occur in concomitance with reduced physical activity and higher intake of processed foods, foods high in calories, unhealthy fats and refined carbohydrates, and exposure to harmful chemicals used in food processing and wrapping^9^—a parallel process known as the nutrition transition.^10^ Together, these shifting patterns result in rising rates of obesity and obesity‐related chronic disease.^11^ Beyond diet and other lifestyle factors, Latino/Hispanic populations have higher prevalence of some genetic polymorphisms that place them at greater risk of obesity‐related disease—a topic reviewed in detail elsewhere.^12^ Thus, in all populations but especially those in Latin America, it is important to identify risk factors and preventive strategies as early as possible.^13^

**FIGURE 1:**
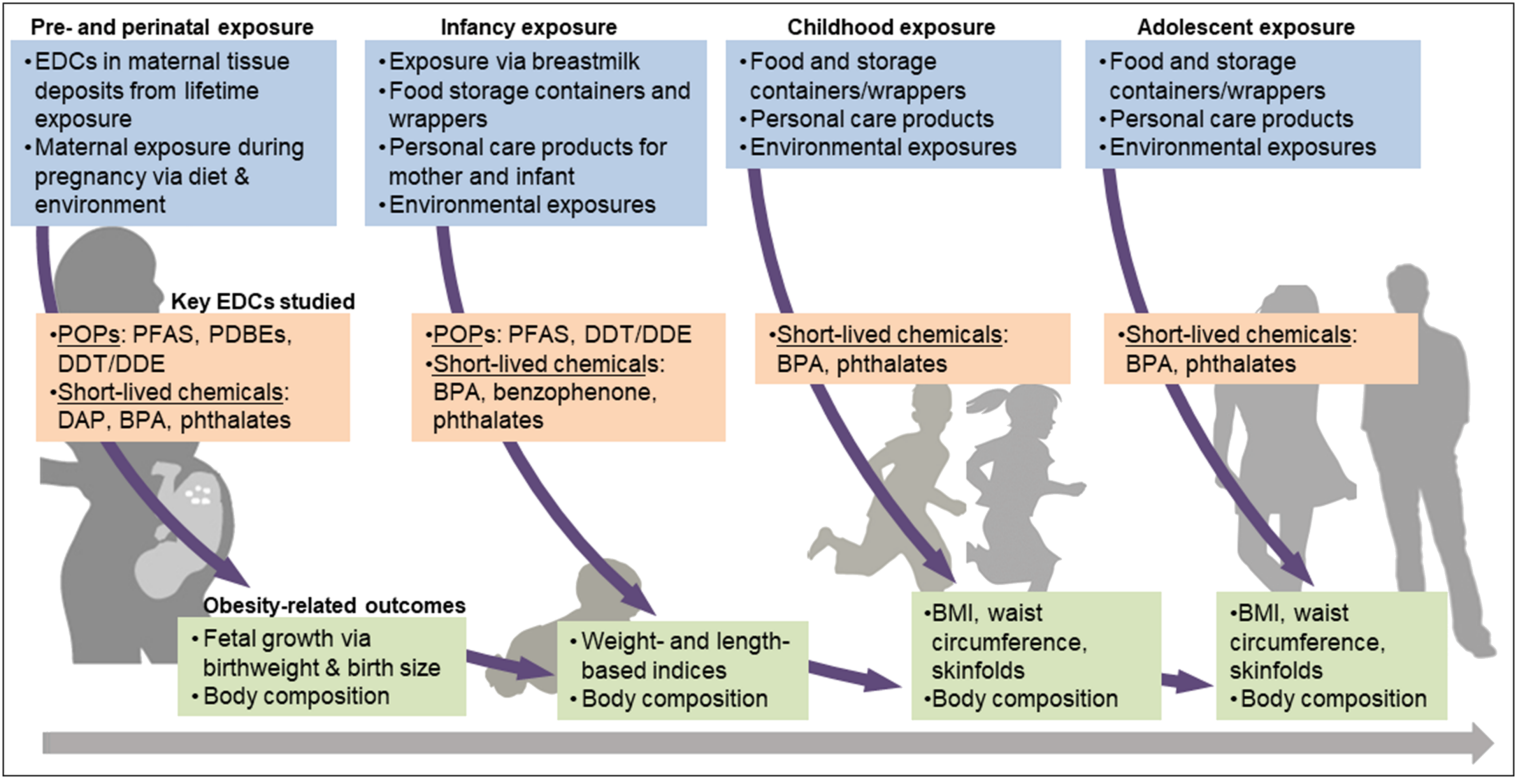
Exposure to obesogenic endocrine disrupting chemicals (EDCs) and childhood obesity across multiple sensitive periods of development

Nearly two decades ago, Baillie‐Hamilton postulated the hypothesis that synthetic chemicals disrupt weight‐control mechanisms and may be a cause of obesity.^14^ These chemicals, known as obesogenic endocrine disrupting chemicals (EDCs) or “obesogens,”^15^, ^16^ have been of increasing interest in recent years. Some EDCs are short‐lived, with half‐lives of days to months (e.g., bisphenol A [BPA] and phthalates), while others are long‐lived and can persist in the environment or in vivo for years (e.g., perfluoroalkyl and polyfluoroalkyl subtances).^17^ Although EDCs were initially developed for beneficial uses such as improvements in agricultural output (e.g., pesticides), enhanced safety of common household items (e.g., flame retardants in upholstery), and personal care (e.g., phthalates in soaps and shampoos), excessive exposure to these chemicals is a negative aspect of industrialization and globalization. Because of their endocrine‐mimicking properties, EDCs can disrupt hormone biosynthesis, lipid metabolism and adipogenesis to promote weight gain and obesity^15^, ^18^, ^19^ through numerous pathways, including but not limited to the following: activating peroxisome proliferator activated receptors,^20^, ^21^ disrupting the biosynthesis and function of sex steroid hormones,^22^, ^23^, ^24^ altering thyroid function,^25^, ^26^ and impacting mechanisms that control appetite and satiety,^20^, ^27^, ^28^ as summarized in Table S1. Moreover, as shown in Figure 1, the impact of EDC exposure may be larger during vulnerable developmental life stages characterized by rapid growth and development, including the in utero period (exposure to the fetus via the mother's environment), infancy (exposure to the infant via the mother during breastfeeding, as well as the infant's own exposure via food/formula containers and household products), early childhood (exposure to the child via diet and other aspects of the environment), and puberty (exposure to the child via diet and environmental exposure).^29^

The objectives of this narrative review are threefold. Our first goal is to review current evidence regarding the effects of early life EDCs exposure on obesity and body composition among youth in Latin America and US youth of Latino/Hispanic descent. Our second goal is to place study findings within a lifecourse framework given the relevance of developmental stages vulnerable to environmental exposures. Our final goal is to discuss avenues for preventive efforts, with a specific focus on policy regulation given the potential for wide‐ranging impact.

## 2 METHODS

This narrative review considers recent epidemiological studies examining the impact of exposure to EDCs on obesity and related measures of fat mass and distribution from birth through adolescence. We focused on original research studies published from January 1, 2010, to January 31, 2020, and restricted articles to human studies of EDC exposure in relation to obesity‐related outcomes. We considered both cross‐sectional and prospective study designs. Given our focus on Latino populations, we only included studies of youth in Latin American countries or those in the United States where Latino or Hispanic participants comprised ≥15% of the sample.

**FIGURE 2:**
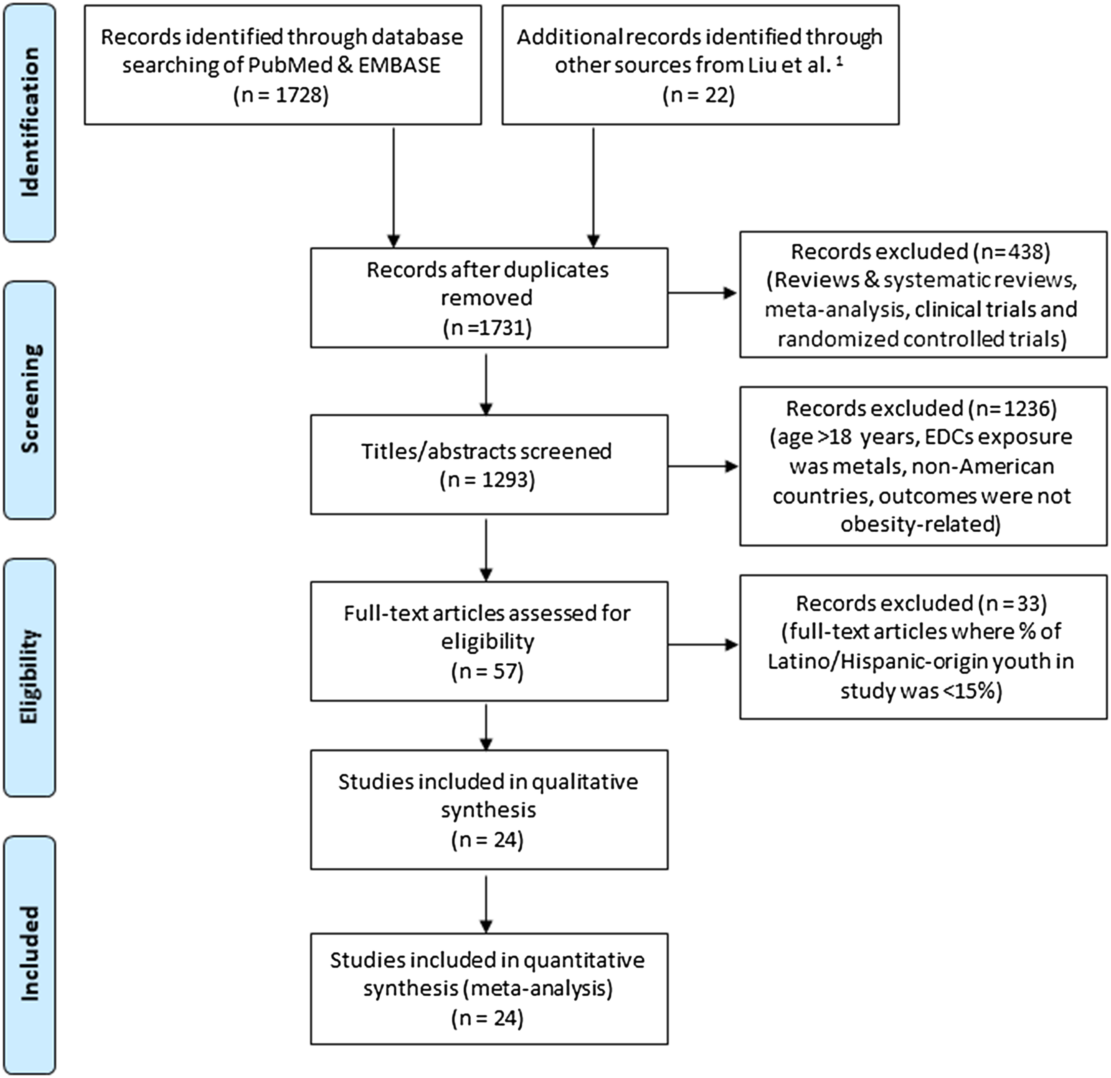
PRISMA 2009 flow diagram

We conducted the literature search for this narrative review in duplicate. In the first step, two independent researchers identified available evidence using a search strategy comprised of separate keywords (i.e., substance and outcome). The search was conducted using Pubmed/Medline and SCOPUS, given our interest in human health and the biomedical sciences, with the following keywords: “environmental obesogen,” “endocrine disrupting compound,” or “polychlorinated biphenyls (PCBs), dichlorodiphenyldichloroethylene (DDE), dichlorodiphenyltrichloroethane (DDT), hexachlorobenzene (HCB), *hexachlorocyclohexane* (HCH), perfluorooctanoic acid (PFOA), perfluorooctane sulfonate (PFOS), perfluoroalkyl substances (PFAS), perfluorinated compound (PFC), polybrominated diphenyl ethers (PBDE), polybrominated biphenyls (PBB), tributyltin (TBT), phthalate, bisphenol A (BPA), organophosphate pesticides (OP), dialkyl phosphate (DAP), diethyl phthalate (DEP), dimethyl phthalate (DMP)” and “infant growth, weight gain, overweight, obesity, birthweight, adiposity, childhood obesity.” We conducted the search in titles, abstract, and full article text in Pubmed and supplemented with additional studies identified through a review led by Liu and Peterson.^30^ After compiling results of the search, we removed duplicates, followed by studies where outcomes were assessed in persons >18 years of age, those that focused on metals as the EDCs of interest given that metals operate through distinct mechanisms from those summarized above (specifically, heavy metals tend to accumulate in organs and disrupt organ function, as opposed to interfering with hormone function),^31^ those that did not take place in the United States or Latin America, those where the outcomes were not obesity‐related, and those where Latino or Hispanic participants comprised <15% of the sample. Abstracts and unpublished studies were not included. Figure 2 shows the PRISMA flow diagram of the literature review pipeline. We organized results according to time period of exposure (prenatal and perinatal periods vs. childhood), followed by life stage of outcome assessment (birth vs. infancy through adolescence) within exposure period, followed by type of EDC within life stage of outcome assessment.

## 3 RESULTS

Using our search strategy, we identified 1731 unique studies. After removal of studies conducted in persons >18 years of age, where the EDCs of interest were metals, where the setting was not in the United States or Latin America, and where the outcomes were not obesity‐related, we considered 57 studies. Of them, 24 met our criterion that the study population contains ≥15% Latino or Hispanic participants.

The study populations were in Mexico, Bolivia, and the United States. The majority (*n* = 16) of studies comprised >90% Latino participants,^32^, ^33^, ^34^ although we note that 10 studies used data from the Center for the Health Assessment of Mothers and Children of Salinas (CHAMACOS) study,^35^, ^36^, ^37^, ^38^, ^39^, ^40^, ^41^, ^42^, ^43^, ^44^ which includes mainly Mexican Americans living in the agricultural region of Salinas Valley in California; and three studies were based in the Early Life Exposure in Mexico to Environmental Toxicants (ELEMENT) Project,^45^, ^46^, ^47^ a birth cohort based in Mexico City. Of the mixed‐race studies, three comprised 50%–70% Latino/Hispanic participants,^48^, ^49^, ^50^ and five included 15%–30% Latino/Hispanic participants.^51^, ^52^, ^53^, ^54^, ^55^ For cohorts with heterogeneous race/ethnic composition (i.e., where the study population is not entirely Latino or Hispanic), the results reported by investigators were not race or ethnicity specific.

Key EDCs of interest in this review included persistent organic pollutants (POPs)—namely, perfluoroalkyl substances (PFAS), dichlorodiphenyltrichloroethane (DDT), dichlorodiphenylxdichloroethylene (DDE), polychlorinated biphenyls (PCB), and polybrominated diphenyl ethers (PBDEs), as well as short‐lived chemicals—namely, phthalates, organophosphorous pesticides (OPs), and phenols. Box 1 includes information on major sources, routes, and extent of human exposure for each EDC class, as well as key associations with obesity‐related outcomes identified from this review.

**Box 1. Key sources, routes of exposure, and obesity‐related physiological effects of endocrine‐disrupting chemicals (EDC)**

**Table:** 

EDC type	Sources and key routes of exposure	Physiological effects
Long‐lived		
Perfluoroalkyl and polyfluoroalkyl substances (PFAS)	Persistent chemicals used in industrial processes for their resistance to heat, oils, stains, grease and water. Major sources included manufacturing/processing facilities and firefighting foams containing these chemicals, which are then released into the air, soil, and water. Other sources of PFAS include food packaging, stain‐ and water‐repellent fabrics and upholstery, non‐stick cookware, and seafood. Key routes of exposure are ingestion, inhalation, and dermal contact.	In utero *exposure is associated with*: • ↓ birthweight and neonatal fat mass • ↑ weight gain during the first 6 months of life • Some evidence for a sex‐specific effect: ↑ adiposity in boys and ↓adiposity in girls at ~5 months
Polybrominated diphenyl ethers (PBDEs)	Persistent organobromine compounds used as flame retardant. Major sources include building materials, motor vehicles, and textiles. PBDEs can be released into the air, water, and soil at places where they are produced or used. Because of low water solubility, PBDEs bind to the sediment and remain in the environment for years. Key routes of exposure are inhalation, ingestion, and dermal contact.	In utero *exposure is associated with*: • ↓ birthweight • ↓ adiposity in girls but ↑adiposity in boys
Dichlorodiphenyltrichloroethane (DDT) and dichlorodiphenyldichloroethylene (DDE)	DDT is a pesticide once widely used to control insects in agriculture and mitigate infectious diseases for which insects are vectors. Due to its detrimental effects to wildlife, DDT was banned in many countries since the early 1970s. DDE is a metabolite of DDT that is found widely in the environment. Key routes of exposure are ingestion and inhalation.	In utero *exposure is associated with*: • ↓ birthweight • ↑ adiposity and overweight/obesity in boys during late childhood & adolescence • Sex‐specific differences in BMI trajectory from birth through 9 years
Short‐lived		
Phthalates	Group of short‐lived chemicals that give plastics flexibility but are also included in solvents, textiles, adhesives, detergents, clothing, and personal care products. Key routes of exposure include ingestion and dermal contact.	In utero *exposure is associated with*: • ↑ birthweight among infants of women with hyperglycemia during pregnancy • ↑ adiposity and overweight/obesity during late childhood and adolescence • Some evidence of sex‐specific associations: ↑ adiposity in girls but ↓ adiposity in boys during mid‐childhood *Concurrent exposure is associated with*: • ↑ adiposity in girls during mid‐childhood
Phenols	Short‐lived chemicals (the most common being bisphenol A) used to make plastics, and included as a disinfectant in household cleaning and consumer products (e.g., mouthwashes, gargles, and throat sprays). Key routes of exposure include ingestion and dermal contact from household products.	In utero *exposure is associated with*: • ↓ adiposity in girls during early and middle childhood *Concurrent exposure is associated with*: • ↑ adiposity in girls but ↓ adiposity in boys during adolescence
Organophosphate pesticides (OPs)	Short‐lived chemicals widely‐used as an insecticide in agriculture, home gardens, and veterinary practice. These insecticides work by damaging enzymes that control nerve signals in insects, and can cause acute neurological effects in humans. Human exposure occurs through ingestion, inhalation, and dermal contact, with higher exposure in agricultural communities.	In utero *exposure is associated with*: • ↑ birthweight among infants of mothers with the non‐susceptible genotype for an enzyme involved in OP detoxification (PON1 gene)

The most commonly assessed obesity‐related outcomes were birthweight (an established marker of the intrauterine environment, an indicator of neonatal adiposity,^56^ and a bellwether for future obesity^57^), body mass index (BMI), and waist circumference. Nine studies obtained direct measures of adiposity via bioelectrical impedance analysis (*n* = 7) or air displacement plethysmography (*n* = 2). Two studies followed participants through puberty (i.e., CHAMACOS and ELEMENT). Prenatal exposure to EDCs was primarily measured in maternal serum and urinary samples during pregnancy (the majority of which were collected during early and/or mid‐pregnancy), and only one study used serum from cord blood (Table 1). In the sections below, we summarize findings regarding exposure to EDCs during each sensitive period of interest within the lifecourse in relation to obesity‐related outcomes in Latino/Hispanic youth. EDCs and their sources, as well as metrics of obesity‐related outcomes reviewed in this paper, are summarized in Figure 1.

**TABLE 1:** Observational studies of the association between prenatal and postnatal exposure to endocrine disrupting chemicals (EDCs) and obesity‐related outcomes in children and adolescents

Reference	Article	Study population & study design	% Latino/Hispanic origin	EDC and timing of exposure	Child age	Outcome	Main results
Persistent organic pollutants (POPs)							
Cupul‐Uicab et al.^32^	Prenatal exposure to the major DDT Metabolite 1,1‐dichloro‐2,2‐bis(p‐chlorophenyl)ethylene (DDE) and growth in boys from Mexico	Study population: 789 mothers and their sons from Chiapas, Mexico. Study design: prospective	100%	Maternal serum DDE and DDT quantified from samples obtained within a day of delivery. Exposures evaluated as continuous variables.	18 months	BMI *z*‐score	NS
Garced et al.^33^	Prenatal dichlorodiphenyldichloroethylene (DDE) exposure and child growth during the first year of life.	Study population: 253 pregnant women in a prospective cohort in Morelos, Mexico. Study design: prospective	100%	Serum levels of DDE quantified from samples obtained during each trimester of pregnancy Exposure evaluated as a continuous variable.	1, 3, 6 and 12 months of age	Weight‐for‐age, weight‐for‐length and BMI‐for‐age *z*‐scores.	NS
Harley et al.^36^	Association of prenatal exposure to polybrominated diphenyl ethers and infant birthweight	Study population: 286 Mexican–American mother‐offspring pairs from the CHAMACOS cohort in Salinas Valley, CA. Study design: prospective	95%	Ten PBDEs congeners quantified from maternal serum samples collected during pregnancy (gestational age 26.1 weeks). Exposures evaluated as continuous variables and categorized in quartiles.	Birth	Birthweight	Certain PBDEs congeners were associated with birthweight: BDE‐47: *β* = −115.4 (95% CI: −229, −1.7) BDE‐99: *β* = −114.4 (95% CI: −224.6, −4.2) BDE‐100: *β* = −121.5 (95% CI: −234.5, −8.5)
Warner et al.^37^	In utero DDT and DDE exposure and obesity status of 7‐year‐old Mexican‐American children in the CHAMACOS cohort	Study population: 270 children from the CHAMACOS cohort in Salinas Valley, CA. Study design*:* prospective	98%	DDT and DDE (o,p*′*‐DDT, p,p*′*‐DDT, and p,p*′*‐DDE) quantified from serum samples collected during pregnancy (gestational age 26 weeks). Exposures evaluated as continuous variables.	2 to 7 years	BMI and WC *z*‐scores, overweight and obesity status	NS
Warner et al.^38^	Prenatal Exposure to Dichlorodiphenyltrichloroethane and Obesity at 9 Years of Age in the CHAMACOS Study Cohort	Study population: 261 children from the CHAMACOS cohort in Salinas Valley, CA. Study design: prospective	98%	DDT and DDE (o,p*′*‐DDT, p,p*′*‐DDT, and p,p*′*‐DDE) quantified from serum samples collected during pregnancy (gestational age 26 weeks) or at delivery. Exposures evaluated as continuous variables.	9 years	BMI and WC *z*‐scores, % fat mass (bioimpedance), and overweight and obesity status	Significant positive associations were observed in boys but not in girls. *Boys* BMI *z*‐score: o,p*′*‐DDT: *β* = 0.34 (95% CI: 0.08, 0.60) p,p*′*‐DDT: *β* = 0.23 (95% CI: 0.03, 0.43) WC *z*‐score: o,p*′*‐DDT: *β* = 0.30 (95% CI: 0.08, 0.52) p,p*′*‐DDT: *β* = 0.23 (95% CI: 0.07, 0.39) Overweight or obesity: o,p*′*‐DDT: OR = 2.5 (95% CI: 1.0, 6.3) p,p*′*‐DDT: OR = 2.1 (95% CI: 1.0, 4.5) p,p*′*‐DDE: OR = 1.97 (95% CI: 0.94, 4.13). Odds of WC ≥ 90th percentile: o,p*′*‐DDT: OR = 1.98 (95% CI: 0.95, 4.11) p,p´‐DDT: OR = 2.05 (95% CI: 1.10, 3.82)
Heggeseth et al.^39^	Detecting associations between early‐life DDT exposures and childhood growth patterns: A novel statistical approach	*Study population:* 249 mother‐offspring pairs from the CHAMACOS cohort in Salinas Valley, CA. Study design: prospective	98.3%	DDT and DDE (o,p*′*‐DDT, p,p*′*‐DDT, and p,p*′*‐DDE) quantified from serum samples collected during pregnancy (gestational age 26 weeks) or at delivery. Exposures evaluated as continuous variables.	2 to 9 years	BMI growth patterns	Four trajectory patterns were identified: (1) linearly increasing; (2) stable and increasing at age 4 to 5; (3) stable and increasing at age 6 to 7; and (4) flat and stable from age 2 until age 9. *Boys:* Higher maternal concentrations of DDT and DDE were associated with higher probability of assignment to groups 2 and 3 versus group 4. *Girls:* An increase in prenatal exposure to DDT was associated with lower probability of being in groups 1 and 3 versus group 4.
Erkin‐Cakmak et al.^40^	In Utero and childhood polybrominated diphenyl ether exposures and body mass at age 7 years: The CHAMACOS study	Study population: 224 mother‐offspring pairs from the CHAMACOS cohort in Salinas Valley, CA. Study design: prospective	>90%	Four PBDEs congeners quantified from serum samples collected during pregnancy (gestational age 26.7 weeks) or at delivery, and from children at a mean of 7 years. Exposures evaluated as continuous variables.	2, 3.5, 5, and 7 years	BMI and WC *z*‐scores, and overweight and obesity status	Prenatal exposure The associations between maternal PBDEs and measures of body mass at 7 years were not significant. However, an effect modification by sex was observed (*p* = 0.04), with significant associations only for girls: *Boys* BMI *z*‐score: Σ4PBDE: *β* = 0.26 (95% CI: −0.19, 0.72) Girls BMI *z*‐score: Σ4PBDE: *β* = −0.41(95% CI: −0.87, −0.05) There were no associations between maternal PBDEs concentrations and repeated measures of body mass from 2 to 7 years, however, there was also evidence of effect modification by sex. Postnatal exposure Children PBDEs concentrations and measures of body mass at 7 years (cross‐sectional analysis): BMI *z*‐score: Σ4PBDE: *β* = −0.44 (95% CI: −0.83, −0.06) WC *z*‐score: Σ4PBDE: *β* = −0.35 (95% CI: −0.66, −0.04) Odds of overweight: Σ4PBDE OR = 0.36 (95% CI: 0.14, 0.94).
Arrebola et al.^34^	Associations of maternal o,p′‐DDT and p,p′‐DDE levels with birth outcomes in a Bolivian cohort	Study population: 200 mother‐offspring pairs from Santa Cruz de la Sierra, Bolivia *Study design:* Cross‐sectional	100%	o,p*′*‐DDT and p,p*′*‐DDE quantified in serum from cord blood. Exposures evaluated as continuous variables.	Birth	Birthweight	Significant associations between pesticides and birthweight were found. p,p*′*‐DDE: *β* = 0.012 (95% CI: 0.003, 0.021) o,p*′*‐DDT: *β* = −0.014, (95% CI: −0.028, −0.001)
Starling et al.^51^	Perfluoroalkyl substances during pregnancy and offspring weight and adiposity at birth: Examining mediation by maternal fasting glucose in the Healthy Start Study	Study population: 628 mother–child pairs from the Healthy Start study in Colorado. Study design: prospective	23%	Eleven PFAS quantified from serum samples collected during pregnancy (median gestational age 27 weeks) Exposures categorized in tertiles.	Birth	Birthweight and % fat mass (air displacement plethysmography)	Certain PFAS were inversely associated with birthweight and adiposity: Birthweight (T3 vs. T1): PFOA: *B* = −92.4 (95% CI: −166.2, −18.5) PFNA: *B* = −92.1 (95% CI: −150.6, −33.6) (high vs. low) % body fat (T3 vs. T1): PFOA: *B* = −0.97 (95% CI:−1.74, −0.20) PFNA: *B* = −0.85 (95% CI:−1.46, −0.24) (high vs. low) PFHxS: *B* = −0.99 (95% CI:−1.75, −0.23) The effect of PFAS on neonatal adiposity was mediated by maternal glucose concentrations.
Warner et al.^41^	Prenatal DDT exposure and child adiposity at age 12: The CHAMACOS study	Study population: 240 mother‐offspring pairs from the CHAMACOS cohort in Salinas Valley, CA. Study design: prospective	98.3%	DDT and DDE (o,p*′*‐DDT, p,p*′*‐DDT, and p,p*′*‐DDE) quantified from serum samples collected during pregnancy (gestational age 26 weeks) or at delivery Exposures evaluated as continuous measures.	2–12 years	WC and BMI *z*‐scores, % fat mass (bioimpedance) and overweight or obesity status	Prenatal DDT and DDE exposure was associated with several adiposity measures in boys at 12 years, but results for girls were non‐significant. *Boys* BMI *z*‐score: o,p*′*‐DDT: β = 0.37 (95% CI: 0.08, 0.65) p,p*′*‐DDT: *β* = 0.26 (95% CI: 0.03, 0.48) p,p*′*‐DDE: *β* = 0.31 (95% CI: 0.02, 0.59). WC *z*‐score: o,p*′*‐DDT: β = 0.31 (95% CI: 0.07, 0.56) p,p*′*‐DDT: *β* = 0.25 (95% CI: 0.05, 0.45) p,p*′*‐DDE: *β* = 0.27 (95% CI: 0.01, 0.53) Risk of Obesity: o,p*′*‐DDT: RR = 1.46 (95% CI: 1.07, 1.97) p,p*′*‐DDT: RR = 1.28 (95% CI: 1.01, 1.64) Risk of WC ≥ 90th percentile: o,p*′*‐DDT: RR = 1.53 (95% CI: 1.12, 2.10) p,p*′*‐DDT: RR = 1.36 (95% CI: 1.05, 1.76) Results from BMI *z*‐score from 2–12 years of age showed similar associations for boys but not for girls.
Buck et al.^53^	Endocrine disruptors and neonatal anthropometry, NICHD fetal growth	Study population: 2106 pregnant women in the NICHD fetal growth studies Study design: prospective	28%	Eleven OCPs, 1 PBB, 9 PBDEs congeners, 44 PCBs, and 11 PFAS were quantified from plasma samples collected during pregnancy (gestational age 10–13 weeks). Exposures evaluated as continuous variables.	Birth	Birthweight	NS
Starling et al.^52^	Prenatal exposure to per‐ and polyfluoroalkyl substances and infant growth and adiposity: the Healthy Start Study	Study population: 415 mother–child pairs from the Healthy Start study in Colorado. Study design: prospective	22%	Eleven PFAS quantified from serum samples collected during pregnancy (median gestational age 27 weeks). Exposures evaluated as continuous variables.	5 months	Weight‐for‐age and weight‐for‐length *z*‐scores, rapid growth from birth, and % fat mass (air displacement plethysmography)	The direction of the associations was different for girls and boys. Among boys, some positive associations were observed: % body fat: PFOA *β* = 1.53 (95% CI: 0.35, 2.71) PFNA *β* = 1.67 (95% CI: 0.56, 2.78) Among girls, some negative associations were observed: Weight‐for‐age *z*‐score: PFOS *β* = −0.26 (95% CI: −0.43, −0.10) PFHxS *β* = −0.17 (95% CI: −0.33, −0.01) In the analyses pooled by sex, MeFOSAA was positively associated with rapid growth: Rapid growth in weight‐for‐age: OR: 2.2 (95% CI: 1.1, 4.3) Rapid growth in weight‐for‐length: OR 3.3 (95% CI: 1.8, 6.2)
Short‐lived chemicals							
Harley et al.^35^	Association of organophosphate pesticide exposure and paraoxonase with birth outcome in Mexican–American women	Study population: 470 mother‐offspring pairs from the CHAMACOS cohort in Salinas Valley, CA. Study design: prospective	98%	Six DAPs metabolites of OPs quantified from urine samples collected twice during pregnancy (gestational age 13.6 and 25.8 weeks, average used) Exposures evaluated as continuous variables.	Birth	Birthweight	There was a significant interaction (*p* = 0.06) between DAPs and PON1 genotype for birthweight. Associations were only statistically significant among children with non‐susceptible PON1 genotype for diethyl phosphate metabolites. Birthweight: DEs: 258.8 g (95% CI: 23.9, 493.6)
Harley et al.^44^	Prenatal and postnatal bisphenol A exposure and body mass index in childhood in the CHAMACOS cohort	Study population: 311 children from the CHAMACOS cohort in Salinas Valley, CA. Study design: prospective	98.7%	BPA quantified in urine samples collected twice during pregnancy (gestational age 13.8 and 26.4 weeks, average used) and collected from children at 5 and 9 years. Exposure as a continuous measure, categorized in tertiles, or <LOD, detectable < the median or > the median.	5 and 9 years	BMI *z*‐score, WC, % fat mass (bioimpedance) and overweight/obesity status	Prenatal exposure Prenatal BPA was associated with some adiposity outcomes at 9 years only among girls. Girls (T3 vs. T1): BMI‐*z* score: *β* = −0.47 (95% CI:−0.87, −0.07) % body fat: *β* = −4.36 (95% CI:−8.37, −0.34) Overweight/obesity: OR = 0.38 (95% CI: 0.16, 0.91) Boys (T3 vs. T1): BMI*z*‐score: *β* = 0.07 (95% CI:−0.31, 0.45) % body fat: *β* = −0.10 (95% CI: −4.61, 4.40) Overweight/obesity: OR = 0.95 (95% CI: 0.36, 2.48) Postnatal exposure BPA at 5 years was not associated with any outcome at 9 years. BPA at 9 years was associated with some adiposity outcomes at 9 years. Boys and girls (detectable > median vs. ≤ LOD): BMI *z*‐score: *β* = 0.55 (95% CI: 0.15, 0.95) WC: *β* = 5.89 (95% CI: 1.19, 10.59) % body fat: *β* = 4.62 (95% CI: 0.26, 8.98) Overweight/obesity: OR = 4.20 (95% CI: 1.60, 11.02).
Maresca et al.^48^	Prenatal exposure to phthalates and childhood body size in an urban cohort	Study population: 424 mother‐offspring pairs from the CCCEH longitudinal birth cohort study's Obesity Project in Manhattan, NY. Study design: prospective	67%	Nine phthalate metabolites quantified from urine samples collected at the 3rd trimester of pregnancy. Exposures grouped in a “DEHP” and a “non‐DEHP” component identified by PCA.	5 and 7 years	BMI *z*‐score, WC, % fat mass (bioimpedance), fat mass index	Some significant interactions for sex were observed (*p* < 0.01). Only among boys, there was a significant association between the “non‐DEHP” component and adiposity outcomes: *5 and 7 years* BMI *z*‐score: Boys: *β* = −0.30 (95% CI: −0.54, −0.06) Girls: *β* = 0.07 (95% CI: −0.18, 0.31) *7 years* % body fat: Boys: *β* = −1.62 (95% CI: −2.91, −0.34) Girls: β = 0.62 (−0.64, 1.88) Fat mass index: Boys: *β* = −0.50 (95% CI: −0.96, −0.04) Girls: *β* = 0.34 (95% CI: −0.15, 0.82) WC: Boys: *β* = −2.02 (95% CI: −3.71, −0.32) Girls: *β* = 1.20 (95% CI:–0.43, 2.84) Results were similar in the models additionally adjusted for urinary metabolite concentrations at child's age 3 and 5.
Buckley et al.^50^	Prenatal exposure to environmental phenols and childhood fat mass in the Mount Sinai Children's Environmental Health Study	Study population: 173 children from the Mount Sinai Children's Environmental Health Study, a cohort study in New York City, NY. Study design: prospective	53.8% (Hispanic or other)	Four phenol biomarkers quantified from urine samples collected at the 3° trimester of pregnancy (gestational age 31.5 weeks). Exposures as continuous measures.	4 to 9 years	% fat mass (bioimpedance)	None of the phenol biomarkers was associated with fat mass, except for benzophenone‐3. For this biomarker, there was evidence of effect modification by child's sex, and the association among girls was marginally significant. Girls: % fat mass: *β* = −1.51 (95% CI: −3.06, 0.01). Boys: % fat mass: *β* = −0.20 (95% CI: −1.69, 1.26).
Buckley et al.^49^	Prenatal phthalate exposures and childhood fat mass in a New York City cohort	Study population: 180 children from the Mount Sinai Children's Environmental Health Study, a cohort study in New York City, NY. Study design: prospective	52.8% (Hispanic or other)	Nine phthalate metabolites quantified in urine samples collected in the 3° trimester of pregnancy (mean gestational age 31.5 weeks). Exposures as continuous measures and categorized in tertiles.	4 to 9 years	Body composition (bioimpedance)	ΣDEHP metabolites (MECPP + MEHHP + MEHP + MEOHP) was associated with body composition. Boys and girls (T3 vs. T1): % body fat: *β* = −3.06 (95% CI: −5.99, −0.09)
Dejerlein et al.^55^	Longitudinal associations of phthalate exposures during childhood and body size measurements in young girls	Study population: 1017 girls from the Breast Cancer and Environment Research Project. Study design: prospective	29%	Nine phthalate metabolites, grouped as low or high molecular weight, quantified in a spot urine sample at ages 6 to 8. Each phthalate group was categorized as low, medium or high.	7 to 13 years	BMI and WC	Low molecular weight phthalates (molar sum of MEP, MBP, and MiBP) were positively associated with BMI and WC trajectories from 7 to 13 years. Girls with high baseline concentrations of these phthalates had greater predicted differences in BMI and WC at 7 years; these became stronger as they aged. For example: BMI and WC at 7 years (high vs. low): BMI: 0.56 (95% CI: −0.02, 1.1) WC: 1.5 (95% CI: −0.38, 3.3) BMI and WC at 13 years (high vs. low): BMI: 1.2 (95% CI: 0.28, 2.1) WC: 3.7 (95% CI: 1.3, 6.5) High molecular weight phthalates (molar sum of MEHP, MEHHP, MEOHP, MECPP, MCPP, and MBzP) were not associated with BMI or WC.
Harley et al.^42^	Association of prenatal urinary phthalate metabolite concentrations and childhood BMI and obesity	Study population: 345 children from the CHAMACOS cohort in Salinas Valley, CA. Study design: prospective	98.3%	Eleven phthalate metabolites quantified from urine samples collected twice during pregnancy (gestational age 14 and 26.9 weeks, average used). Exposures as continuous measures and categorized in quartiles.	5 to 12 years	BMI and WC *z*‐scores, % fat mass (bioimpedance) and overweight/obesity status.	Several phthalate metabolites were associated with adiposity outcomes at multiple ages. The results at 12 years are presented: *MEP, boys and girls* BMI *z*‐score: MEP: β = 0.10 (95% CI: 0.04, 0.16) WC *z*‐score: MEP: *β* = 0.10 (95% CI: 0.04, 0.16) % body fat: MEP: *β* = 1.0 (95% CI: 0.3, 1.7) Overweight/obesity: MEP (continuous): OR = 1.3 (95% CI: 1.1, 1.5) MEP (Q4 vs. Q1): OR = 2.0 (95% CI: 1.0, 3.9) *Other metabolites, boys and girls except otherwise indicated* Overweight/obesity: MBP (continuous): OR = 1.2 (95% CI: 1.0, 1.4) MBP (continuous): OR = 1.4 (95% CI: 1.1, 1.8)‐*only in boys* MBP (Q4 vs. Q1): OR = 2.1 (95% CI: 1.1, 4.2) ΣDEHP (continuous): OR = 1.3 (95% CI: 1.0, 1.6) ΣDEHP (Q4 vs. Q1): OR = 2.2 (95% CI: 1.1, 4.5) MBzP (continuous): OR = 1.2 (95% CI: 1.0, 1.4) MBzP (Q4 vs. Q1): OR = 1.9 (95% CI: 0.9, 3.7) BMI and WC *z*‐scores: Some associations were observed for MBP, MiBP, MBzP, and ΣDEHP but these were less consistent and stronger at younger ages.
Yang et al.^46^	Bisphenol A and phthalates in utero and in childhood: association with child BMI *z*‐score and adiposity	Study population: 249 children from the ELEMENT cohort in Mexico City. Study design: prospective	100%	BPA and 9 phthalate metabolites quantified from urine samples collected in the 3rd trimester of pregnancy and from children at 8 to 14 years. Exposures as continuous measures.	8 to 14 years	BMI *z*‐score, WC and sum of skinfold thickness (triceps and subscapular).	Associations between BPA and phthalate metabolites and adiposity outcomes were specific for exposure timing (prenatal vs. postnatal) and sex (only for postnatal exposure). Prenatal exposure BMI *z*‐score: MBzP: *β* = −0.21 (95% CI: −0.41,‐0.02) Postnatal exposure WC: MEHP: *β* = −1.86 (95% CI: −3.36,−0.35) Sum of skinfold thickness: MEHP: *β* = −2.08 (95% CI: −3.80,−0.37) Evidence of effect modification by sex for some metabolites: *Boys* BMI *z*‐score: MEOHP: *β* = −0.26 (95% CI: −0.51,−0.005) WC: MECPP: *β* = −2.13 (95% CI: −4.22,−0.04) MEHHP: *β* = −2.02 (95% CI: −4.02,−0.03) Sum of skinfold thickness: MEHP: *β* = −2.95(95% CI: −5.08,‐0.82) *Girls* Sum of skinfold thickness: BPA: *β* = 3.47(95% CI: 0.05, 6.40)
Yang et al.^45^	Exposure to bisphenol A and phthalates metabolites in the third trimester of pregnancy and BMI trajectories	Study population: 249 children from the ELEMENT cohort in Mexico City. Study design: prospective	100%	BPA and 9 phthalate metabolites quantified from urine samples collected in the 3rd trimester of pregnancy (mean gestational age 34.1 weeks). Exposures categorized in tertiles.	Birth to 14 years	BMI trajectories	Certain phthalates metabolites were associated with BMI trajectories. Girls: The third tertile of MECPP exposure predicted the highest BMI trajectory by adolescence. Boys: The highest BMI trajectory by 14 years was predicted by exposure to the first tertile of MiBP and MBzP, and to the second tertile of MEHP and MEHHP.
Bowman et al.^47^	Phthalate exposures, DNA methylation and adiposity in Mexican children through adolescence	Study population: 223 mother‐offspring pairs from the ELEMENT cohort in Mexico City. Study design: prospective	100%	Nine phthalate metabolites quantified from urine samples collected at each trimester of pregnancy, and at 8 to 14 years in children. Exposures as continuous measures.	8 to 14 years and again at 9 to 17 years	BMI *z*‐score, WC and sum of skinfold thickness (triceps and subscapular).	Associations between phthalate metabolites and adiposity outcomes were specific by sex‐and exposure timing. Prenatal exposure (outcomes 8 to 17 years) *Girls* BMI *z*‐score: MBP: *β* = 0.25 (95% CI: 0.03, 0.46) (1st trimester). MiBP: *β* = 0.28 (95% CI: 0.12, 0.45) (first trimester). Sum of skinfold thickness: MiBP: *β* = 3.41 (95% CI: 1.50, 5.31) (first trimester). MBzP: *β* = −2.53 (95% CI: −4.78, −0.28) (second trimester). WC: MiBP: *β* = 2.33 (95% CI: 0.86, 3.8) (first trimester). *Boys* BMI *z*‐score: MBzP: *β* = 0.25 (95% CI: 0.01, 0.49) (2nd trimester). WC: MBzP: *β* = 2.11 (95% CI: 0.27, 3.95) (second trimester). Postnatal exposure (outcomes 9 to 17 years, no associations found for girls) *Boys* Sum of skinfold thickness: MBzP: *β* = −2.43 (95% CI: −4.69, −0.17) WC: MBzP: *β* = −1.91 (95% CI: −3.64, −0.19) There was suggestive evidence of a mediation effect by DNA methylation but the results were not statistically significant.
Heggeseth et al.^43^	Heterogeneity in childhood body mass trajectories in relation to prenatal phthalate exposure	Study population: 335 children from the CHAMACOS cohort in Salinas Valley, CA. Study design: prospective	>90%	Eleven phthalate metabolites quantified from urine samples collected twice during pregnancy (gestational age 14 and 26.9 weeks, average used). Exposures as continuous measures.	2 to 14 years	BMI and BMI *z*‐score trajectories over childhood using different methods	Using different methods, certain phthalates metabolites were associated with BMI trajectories in boys and girls. We describe selected results. MEP was the most prevalent phthalate metabolite and its associations with BMI were different for boys and girls. *Boys* MEP had a positive and linear association with BMI at several points throughout childhood. *Girls* MEP had a non‐linear association starting at age 7. Some metabolites were associated (nonsignificant, *p* < 0.10) with increased or decreased probability ratios of being in a specific class of BMI trajectory (Classes 1–3) vs. the reference (Class 4). The significant results for trajectories using raw BMI are presented: *Boys* MECPP (Class 3 vs. ref.): 1.38 (95% CI: 0.97, 1.96) *Girls* MCNP (Class 3 vs. ref.): 0.73 (95% CI: 0.51, 1.04) MCOP (Class 3 vs. ref.): 0.70 (95% CI: 0.49, 1.02) MEHP (Class 2 vs. ref.): 1.45 (95% CI: 0.99, 2.12) MEHHP (Class 2 vs. ref.): 1.42 (95% CI: 0.96, 2.10) MECPP (Class 2 vs. ref.): 1.51 (95% CI: 0.98, 2.34) MEOHP (Class 2 vs. ref.): 1.42 (95% CI: 0.95, 2.10) Description of BMI trajectories: Class 1, steep linear increase in BMI; Class 2, increased steeply in childhood but leveled off (girls) or decreased (boys) around puberty; Class 3, moderately increasing pattern; Class 4, relatively stable pattern.
Noor et al.^54^	Pregnancy phthalate metabolite concentrations and infant birthweight by gradations of maternal glucose tolerance	Study population: 350 mother‐infant pairs from the LIFECODES pregnancy cohort in Boston, MA. Study design: prospective	15%	Nine phthalate metabolites quantified from urine samples collected 4 times during pregnancy (gestational age 9.9, 17.9, 26.1 and 35.3 weeks, average used). Exposures categorized in quartiles	Birth	Birthweight	Phthalate metabolites were not associated with birthweight. In the analysis stratified by maternal glucose levels, an association was observed among women with high glucose. Maternal glucose ≥140 mg/dl: MCPP (Q4 vs. Q1): *β* = 569.22 g (95% CI: 14.1, 1178.2).

Abbreviations: BMI, body mass index; BPA, bisphenol A; CCCEH, Columbia Center for Children's Environmental Health; CHAMACOS, Center for the Health Assessment of Mothers and Children of Salinas; DAPs, dialkyl phosphates; DDE, dichlorodiphenyldichloroethylene; DDT, dichlorodiphenyltrichloroethane; DEs, diethyl phosphate metaboilites; DEHP, di(2‐ethylhexyl) phthalate; ELEMENT, Early Life Exposures in Mexico and Environmental Toxicology; LOD, limit of detection; MBP, mono‐*n*‐butyl phthalate; MBzP, monobenzyl phthalate; MCNP; mono (carboxynonyl) phthalate; MCPP, mono(3‐carboxypropyl) phthalate; MCOP, mono (carboxyoctyl) phthalate; MECPP, mono(2‐ethyl‐5‐carboxypentyl) phathalate; MeFOSAA, 2‐(N‐methyl perfluorooctane sulfonamido) acetate; MEHHP, mono(2‐ethyl‐5‐hydroxyhexyl) phthalate; MEHP, mono(2‐ethylhexyl) phthalate; MEOHP, mono(2‐ ethyl‐5‐oxohexyl) phthalate; MEP, monoethyl phthalate; MiBP, mono‐isobutyl phthalate; NICHD, National Institute of Child Health and Human Development; NS, nonsignificant; OCPs, organochlorine pesticides; OPs, organophosphorus pesticides; PBB, polybrominated biphenyl; PBDEs, polybrominated diphenyl ethers; PCA, principal component analysis; PCBs, polychlorinated biphenyls; PFAS, per‐and‐ polyfluoroalkyl substances; PFHxS, perfluorohexane sulfonate; PFNA, perfluorononanoate; PFOA, perfluorooctanoate; PFOS, perfluorooctane sulfonate; PON1, paraoxonase 1; POPs, persistent organic pollutants; WC, waist circumference.

### 3.1 EDC exposure during the prenatal and perinatal periods

The majority of the studies focused on EDC exposure during the prenatal and perinatal periods, with exposure assessment typically occurring during early or mid‐pregnancy. While exposures around the time of conception and during pregnancy occur within the prenatal period, and those that occur immediately before or after birth take place within the perinatal period, we discuss findings for these two periods together given wide variability in the week of gestation at which EDC exposure was assessed, and the fact that assessments of exposure via maternal tissues in late pregnancy and at delivery may also reflect EDC exposure earlier in pregnancy. We summarize key findings below.

#### 3.1.1 Prenatal and perinatal exposure ➔ adiposity at birth

We identified six studies that assessed prenatal and perinatal EDC exposure in relation to outcomes at birth. Birthweight was the most widely used indicator of adiposity, which makes sense given the wealth of literature linking both low^58^, ^59^, ^60^, ^61^ and high birthweight^57^ to obesity and obesity‐related outcomes later in life.^30^ Only one study (the Healthy Start Study) had data on directly measured body composition via air‐displacement plethysmography.^51^

The majority of studies reported inverse associations of prenatal and perinatal exposure to EDCs with neonatal adiposity, even after adjustment for key covariates like maternal age, pre‐pregnancy weight status, glycemia during pregnancy (e.g., mid‐pregnancy glucose levels and gestational diabetes status), smoking habits, gestational weight gain, gestational age at delivery, and infant sex. We discuss findings with respect to specific classes of EDCs, below.

##### PFAS

Among 628 mother‐infant pairs in the Healthy Start Study, a Colorado‐based pre‐birth cohort comprising 23% Hispanic participants, Starling et al.^51^ reported that concentrations of two PFAS, perfluorooctanoate (PFOA) and perfluorononanoate (PFNA), measured in maternal serum collected at median 27 weeks gestation were inversely related to birthweight (*β* for highest tertile [T3] vs. lowest tertile [T1] of PFOA = −92.4 [95% confidence interval, CI: −166.2, −18.5] g; *β* for above vs. below the median for PFNA = −92.1 [95% CI: −150.6, −33.6] g).^51^ The authors noted similar associations for PFOA, PFNA, and perfluorohexane sulfonate (PFHxS) in relation to directly measured % fat mass assessed (e.g., *β* for T3 vs. T1 of PFOA was −0.97 [95% CI: −1.74, −0.20]%). These findings suggest that some PFAS (e.g., PFOA) may influence fetal accrual of adiposity, while others affect overall size of the newborn (e.g., PFNA).

##### PBDEs

In 286 participants of the CHAMACOS study comprising Mexican–Americans living in the agricultural region of Salinas Valley in California, maternal serum levels of PBDEs assessed at 26 weeks gestation were inversely associated with birthweight. Each 10‐fold increase in BDE‐47 concentration corresponded to a 115 (95% CI: 2, 228)‐g decrement in birthweight, with similar findings for BDE‐99 and BDE‐100. However, the authors noted that after adjustment for gestational weight gain, the results were no longer statistically significant.^36^

##### DDT and DDT

We found one study that assessed prenatal and perinatal exposure to DDT and DDE via cord blood. Among 200 mother–child pairs in the highly exposed birth cohort located in Santa Cruz de la Sierra of Bolivia where metabolites of the POPs DDT were detected in more than 80% of cord blood samples, Arrebola et al. found that higher cord blood levels of *o,p′*‐DDT corresponded with lower birthweight (−0.014 [95% CI: −0.028, −0.001] units log birthweight per 1 unit *o,p′*‐DDT), whereas *p,p′*‐DDE was related to higher birthweight (0.012 [95% CI: 0.003, 0.020 units log birthweight per 1 unit as *o,p′*‐DDT) in mutually adjusted models that also accounted for key covariates: maternal age, parity, smoking habits, gestational weight gain, BMI, and gestational age at delivery.^34^ Interestingly, despite being associated with higher birthweight, *p,p′*‐DDE was related to shorter gestation length (−0.004 [95% CI: −0.008, −0.001] units of log gestational age per 1 unit *p,p′*‐DDE),^34^ suggesting that long‐term DDT exposure may promote more rapid fetal growth at any given gestational age, and in spite of shorter gestation length.

In contrast, in an analysis of 253 mother–infant dyads in Morelos, Mexico, Garced et al. did not find any association of maternal serum DDE concentrations during any trimester of pregnancy with anthropometry at birth (weight, length, head circumference, and arm and thigh length), or with growth from birth through 1 year of age.^33^ The discrepancy in findings could be due to differences in the biospecimen type; Arrebola et al. analyzed cord blood whereas Garced et al. analyzed maternal serum, the latter of which may not represent true fetal exposure due to protective functions of the placenta. Other possible reasons for differences in findings include differences in level of exposure (though difficult to assess directly given the different biospecimen types), variability in covariate adjustment (Garced et al. included a number of lifestyle characteristics, like maternal diet during pregnancy, that were not accounted for by Arrebola et al.), and or other differences in the study populations (Bolivian vs. Mexican participants).

##### POPs mixtures

In addition to the above‐mentioned studies that document inverse associations of prenatal and perinatal POPs exposure on neonatal adiposity, one study reported null findings. In the National Institute of Child and Human Development Fetal Growth Studies, a large cohort of 2106 healthy mother–infant dyads recruited from 12 sites across the United States of which over 25% are of Latino/Hispanic origin, Buck Louis et al. explored associations of 76 POPs in maternal serum during the first trimester of pregnancy, including PBDEs, polybrominated biphenyls (PBB), and PFAS with neonatal anthropometry.^53^ The authors found no relationship between these chemicals and neonatal adiposity, although they noted inverse associations of PFAS with arm and thigh length. These findings suggest that bone length at birth may be a sensitive marker of early intrauterine EDC exposure, which is relevant to obesity and its sequelae given that shorter stature later in life has been linked to an adverse metabolic profile.^62^

##### Phthalates

A few studies have examined associations with respect to short‐lived EDCs. A study of 350 participants in the Lifecodes pregnancy cohort in Boston, of which 15% of participants are Latino/Hispanic, found that average concentrations of mono‐(3‐carboxylpropyl) phthalate (MCPP) across gestation was associated with higher birthweight among mother–child pairs for which the mother had post‐50‐g glucose challenge test glucose level was ≥140 mg/dl),^54^ suggesting a potential synergistic interaction between some phthalates and maternal hyperglycemia.

##### Organophosphate pesticides

Following a study in CHAMACOS showing generally null associations of organophosphate pesticide exposure with birth size,^63^ Harley et al. led a gene x environment study among 470 mother–infant dyads to explore effect modification to the relationship between organophosphate pesticide exposure as measured by nonspecific dialkyl phosphate (DAP) metabolites in maternal urine, and birth outcomes by paraoxonase 1 (PON1), a gene that regulates expression of the paraoxonase enzyme involved in detoxification of organophosphorus pesticides.^35^ The authors found that higher maternal urinary DAP concentrations were associated with shorter gestation length, particularly among those with the susceptible PON1 genotype. They also noted that maternal urinary levels of DAP metabolites were positively associated to birthweight, but only among infants with the non‐susceptible PON1 genotype. Such findings are unexpected but may be due to the fact that in non‐susceptible women, high urinary DAP concentrations may be an indicator of more rapid metabolism and detoxification of organophosphorus pesticides, as opposed to high exposure.

#### 3.1.2 Prenatal and perinatal exposure➔ adiposity from infancy through adolescence

Due to the relatively small literature on prenatal/perinatal EDC exposure and adiposity beyond birth, we summarize the evidence on obesity outcomes from infancy through adolescence together. We identified three studies for which outcomes were assessed during infancy (<2 years of age).^32^, ^33^, ^52^ The other 15 studies involved participants across a wide range of age: Most studies (*n* = 8) focused on early and middle childhood from 2 to approximately 9 years of age,^37^, ^38^, ^39^, ^40^, ^44^, ^48^, ^49^, ^50^ some (*n* = 4) eclipsed the peripubertal transition from late childhood into adolescence age 5 through 18 years,^42^, ^46^, ^47^, ^55^ and three studies spanned 2 years through 12–14 years^41^, ^43^, ^45^ (nota bene, Yang et al. assessed associations of EDCs with longitudinal growth for individual children from 2 through 14 years of age, as opposed to children of differing age groups).

Common anthropometric indicators of adiposity include age‐ and sex‐standardized weight and length based indices (weight‐for‐age, weight‐for‐length, and BMI‐for‐age), waist circumference, and skinfold thicknesses. Additionally, at older ages (i.e., middle childhood onward), some cohorts collected data on body composition via air displacement plethysmography and bioimpedance analysis.

##### PFAS

In the previous section, we reported findings of Starling et al.^51^ of an inverse relationship between maternal serum PFAS and offspring birthweight. In a follow‐up study comprising 415 mother–infant pairs,^52^ the investigators found that several PFAS were related to higher adiposity at 5 months, as well as with rapid weight gain from birth to 5 months in a sex‐specific fashion. Among male infants, maternal serum PFOA and PFNA were positively associated with higher % fat mass at 5 months, ranging 1.5%–1.7% per 1‐unit natural‐log (ln)‐transformed ug/ml of a given chemical. Among females babies, maternal serum levels of perfluorooctane sulfonate (PFOS) and PFHxS were associated with lower weight‐for‐age *z*‐score at 5 months (PFOS: *β* = −0.26 [95% CI − 0.43, −0.10] SD per ln‐ng/ml; PFHxS: *β* = −0.17 [95% CI: −0.33, −0.01] SD per ln‐ng/ml PFHxS). In analyses pooled by sex, 2‐(*N*‐methyl‐perfluorooctane sulfonamido) acetate above versus below the limit of detection was associated with greater odds of rapid adiposity gain, as indicated by change in weight‐for‐age or weight‐for‐length equivalent to ≥0.67 *z*‐scores between birth and 5 months of age (odds ratio [OR] = 2.2 [95% CI 1.1, 4.3] for weight‐for‐age; OR = 3.3 [95% CI 1.8, 6.2] for weight‐for‐length).^52^ Such findings indicate that while PFAS are inversely related to birthweight, which includes both fat and fat‐free mass, these chemicals may be associated with greater fat accrual during early infancy in a sex‐specific manner.

##### PBDEs

In 224 CHAMACOS participants, Erkin‐Cakmack et al. found that maternal serum PBDEs concentrations during late pregnancy (~27 weeks) and delivery were associated with higher BMI *z*‐score among boys (*β* = 0.26 [95% CI: −0.19, 0.72]) and lower BMI *z*‐score among girls (*β* = −0.41 [95% CI: −0.87, −0.05]) at 7 years of age after controlling for maternal age, education, pre‐pregnancy BMI, gestational weight gain, years of residence in the United States, poverty during pregnancy, offspring gestational age at delivery, duration of breastfeeding, and child's fast food and soda consumption.^40^

##### DDT and DDE

Heggeseth et al.^39^ characterized four distinct BMI growth trajectory classes for 249 participants from the CHAMACOS cohort and then examined sex‐specific associations of DDT and DDE with odds of membership in each trajectory class. The investigators found that higher maternal serum concentrations of DDT and DDE during pregnancy were associated with a BMI gain pattern characterized by a stable increase from 2 to 5 years, followed by a rapid increase in BMI gain from 5 to 9 years of age in boys.^39^ In girls, higher prenatal DDT exposure was associated with stable growth from 2 to 9 years of age.^39^ Moreover, while DDT and DDE exposure was not related to obesity status at 7 years of age in this cohort,^37^ investigators noted a sex‐specific effect of these chemicals on adiposity at later ages among boys only. Specifically, starting at age 9 years, the odds of being overweight or obese doubled per 10‐fold increase in prenatal concentrations of DDT and DDE in boys.^38^ By 12 years of age, prenatal exposure to DDT and DDE was associated not only with overweight/obesity but also with continuous BMI *z*‐score and waist circumference in boys, even after accounting for pubertal status.^41^ On the other hand, in a highly exposed population in Chiapas, Mexico, Cupul‐Uicab et al. found no association of DDE exposure with BMI growth patterns from birth through 18 months in 789 children.^32^ These null findings could be due to the fact that health effects of toxicants may not become apparent until middle to late childhood.

##### Phthalates

Of the different classes of toxicants reviewed herein, prenatal exposure to phthalates received the most attention, although the evidence across study populations is inconsistent. These discrepancies in findings with respect to phthalates and phenols across individual studies may arise from differences in nativity of the study populations, as well as challenges of assessing exposure to short‐lived chemicals.

In CHAMACOS, a study of 345 participants found that maternal urinary concentrations of the low‐molecular weight phthalates diethyl phthalate (DEP) and dibutyl phthalate (DBP), and the high‐molecular weight di(2‐ethylhexyl) phthalate (DEHP) during pregnancy, were positively associated with BMI *z*‐score, waist circumference *z*‐score, and percent body fat during childhood, with particularly strong associations with overweight/obesity status at 12 years of age (OR = 1.3 [95% CI: 1.1, 1.5] per twofold increase in DEP; OR = 1.2 [95% CI: 1.0, 1.4] per twofold increase in DBP; and OR = 1.3 [95% CI:1.0, 1.6] per twofold increase in DEHP),^42^ as well as with continuous BMI *z*‐score through 14 years of age.^43^ We note that in these analyses, the investigators did not adjust for pubertal status as it could be on the causal pathway between EDC exposure and weight status or growth.

In contrast to findings in CHAMACOS, a study of 326 mother–child pairs from the Columbia Center for Children's Environmental Health (CCCEH) Obesity Project (67% of participants are Dominican) found inverse associations of maternal phthalate exposure and offspring adiposity. Higher levels of maternal urinary mono‐isobutyl phthalate (MiBP), mono‐*n*‐butyl phthalate (MBP), monobenzyl phthalate (MBzP), monoethyl phthalate (MEP), and MCPP operationalized as a single latent variable (via principal components analysis) was associated with lower BMI *z*‐score (*β* = −0.30 [95% CI: −0.54, −0.06]), and % body fat (*β* = −1.62 [95% CI: −2.91, −0.34]), and smaller waist circumference (*β* = −2.02 [95% CI: −3.71, −0.32] in boys.^48^

Among 249 participants from the ELEMENT Project, Yang et al.^46^ reported that maternal urinary concentration of MBzP during the third trimester was inversely associated with BMI *z*‐score at age 8–14 years (*β* = −0.21 [95% CI: −0.41, −0.02]). In a subsequent study that involved 223 of 249 participants in Yang et al.'s analysis, Bowman et al.^47^ explored potential mediation of the effects of trimester‐specific phthalate exposure on adiposity trajectories from 8–14 to 9–17 years of age and found both trimester‐ and sex‐specific associations. In girls, exposure during the first trimester appeared to be more important than later in pregnancy. Specifically, maternal urinary MBP and MiBP concentrations during the first trimester were positively associated with BMI *z*‐score across the two time points (*β* = 0.25 [95% CI: 0.03, 0.46] per 1 unit natural log‐transformed MBP; *β* = 0.28 [95% CI: 0.12, 0.45] per 1 unit natural log‐transformed MiBP). On the other hand, mid‐pregnancy exposure appeared to be most relevant for boys; that is, higher maternal urinary MBzP concentrations in the second trimester were associated with higher BMI *z*‐score (*β* = 0.25 [95% CI: 0.01, 0.49] per 1 unit ln‐MBzP) and waist circumference (*β* = 2.11 [95% CI: 0.27, 3.95] cm per 1 unit ln‐MBzP).^47^

In an analysis of 180 participants from the Mount Sinai Children's Environmental Health Study (52.8% Latino/Hispanic participants), prenatal exposure to phthalates was not associated with adiposity at 4–9 years of age, with the exception of an inverse relation of tertiles of maternal urinary concentrations of the sum of DEHP metabolites (∑DEHP) with % fat mass measured by bioimpedance analysis at 4–9 years of age (*β* for T3 vs. T1 of ∑DEHP = −3.06% [95% CI: −5.99%, −0.09%] for both boys and girls).^49^

##### Phenols

In another study of participants (*n* = 173) from the Mount Sinai Children's Environmental Health Study, concentrations of benzophenone‐3 (a naturally occurring phenol occurring in flowering plants, but used in lotions and cosmetics for its ultraviolet ray absorbing properties^64^) in maternal urine during pregnancy were negatively associated with % body fat in 4‐ to 9‐year‐old girls (*β* = −1.51 [95% CI: −3.06, 0.01], but not in boys.^50^ In a similar vein, a study of 311 children from the CHAMACOS study observed that average urinary BPA concentrations across two measurements in pregnancy were negatively associated with BMI *z*‐score and % body fat in female offspring only at 9 years of age (BMI *z*‐score: *β* = −0.47, 95% CI: −0.87, −0.07]; % fat mass: *β* = −4.36% [95%CI: −8.37%, −0.34%] for T3 vs. T1 of maternal urinary BPA concentrations).^44^ On the other hand, among 249 youth from the ELEMENT Project, no associations were found between prenatal BPA concentrations and offspring BMI *z*‐score during middle to late childhood,^46^ or with respect to BMI trajectories across the peripubertal transition.^45^

### 3.2 EDC exposure during childhood

We identified four studies from three cohorts that characterized exposure to EDCs during childhood and/or adolescence (roughly defined as age 3 through 15 years) and related EDC exposure to adiposity outcomes. All four of these studies focused on BPA and phthalate exposure.

The first study was a longitudinal investigation of girls in The Breast Cancer and Environment Research Program. In this study, Deierlein et al. measured urinary concentrations of nine phthalate metabolites of 1017 girls aged 6–8 years at enrollment. The authors assessed associations of the phthalates at baseline (categorized as low, medium, and high) with change in BMI, height, and waist circumference during 7 years of follow‐up. The primary finding was that girls with the highest molar sum of the low‐molecular‐weight phthalates MEP, MBP, and MiBP (referred to as ΣDBP) exhibited a greater increase in BMI and waist circumference than those with the lowest concentrations of ΣDBP.^55^

The second study, among 311 CHAMACOS participants, found that urinary concentrations of BPA at 5 years of age was not related to adiposity at 5 or 9 years of age.^44^ However, at 9 years of age, higher concurrent BPA exposure, dichotomized as above the median vs. below the level of detection, was associated with higher BMI *z*‐score (*β* = 0.55 [95% CI: 0.15, 0.95]), waist circumference (*β* = 5.89 cm [95% CI: 1.19, 10.59]) and % fat mass [*β* = 4.62% [95% CI: 0.26%, 8.98%]). In this cohort, data were also available for four PBDEs measured at age 7 years in 224 participants, the sum of which (Σ4PBDE) exhibited a negative association with concurrent BMI *z*‐score (*β* = −0.44 [95% CI: −0.83, −0.06] per 1‐unit increment in Σ4PBDE) and waist circumference *z*‐score (*β* = −0.35 [95% CI: −0.66, −0.04] per 1‐unit increment in Σ4PBDE).^40^

Finally, one cross‐sectional analysis in the ELEMENT Project linked urinary concentrations of BPA and phthalates to adiposity indicators at 8–14 years of age. Yang et al. found that higher urinary BPA was associated with higher skinfold sum among 117 girls (*β* = 3.47 mm [95%CI: 0.05, 6.40] per 1‐unit ln‐BPA).^46^ In boys (*n* = 132), the authors detected an inverse relationship between mono(2‐ethylhexyl) phthalate (MEHP) and skinfold thicknesses (*β* = −2.95 [95% CI: −5.08, −0.82] per 1‐unit ln‐MEHP). Subsequently, Bowman et al. led a follow‐up study that included a repeat assessment of adiposity measures 3 years later in 223 participants and found an inverse association of MBzP with average skinfold thickness (*β* = −2.43, 95% CI: −4.69,−0.17) and waist circumference (*β* = −1.91 cm, 95% CI: −3.64,−0.19) among boys (*n* = 109), but no associations in girls (*n* = 114).^47^

## 4 DISCUSSION

### 4.1 Summary of findings

Here, we build upon existing reviews^11^, ^65^ that summarize the influence of environmental and behavioral factors to obesity in Latino/Hispanic youth by honing in on the specific contributions of EDCs to obesity‐related outcomes in this vulnerable population. This is a topic of great importance given that identification of key chemicals that are consistently associated with excess adiposity in Latino/Hispanic youth across research publications and study populations may inform preventive action.

Our review identified 24 published studies in the last decade that examined associations of early life exposure to EDCs with obesity‐related outcomes in Latino/Hispanic youth in the United States or Latin America. Over half (*n* = 13) of these studies were based on two cohorts of Mexican/Mexican–Americans (CHAMACOS in the Salinas Valley of California with >90% Latino participants, and ELEMENT in Mexico City with 100% Latino participants). In addition to studies based on these two cohorts, on study in Cruz de la Sierra in Bolivia,^34^ and two additional studies in Mexico (Chiapas^32^ and Morelos^33^) had 100% Latin American participants. The remainder of the studies (*n* = 8) comprised mixed race populations, with 15%–70% Latino/Hispanic participants, though the investigators did not report race/ethnicity‐specific estimates, making it difficult to directly compare estimates across studies. Regardless, we noted some general trends with respect to EDC exposure, adiposity outcomes, and research findings.

The current literature focuses largely on EDC exposure during the prenatal and perinatal periods, which is likely related to numerous animal models indicating the importance of this timeframe as a sensitive period for environmental exposures.^18^, ^66^, ^67^ We also noted that DDT/DDE, organophosphate pesticides, and phthalates have received quite a bit of attention, which makes sense given the increased use of these chemicals in industrializing and agricultural countries such as those in Latin America.^68^

During the childhood and adolescent life stages, the majority of studies focused on exposure during the prenatal and perinatal periods. The chemical of greatest interest were nonpersistent chemicals like BPA and phthalates, which may become more relevant in older children who regularly consume packaged snacks and beverages, which are major sources of BPA and phthalates. Older children also use personal care products that are fraught with such chemicals (e.g., deodorants, cosmetics, and hair products).

With respect to health outcomes, most studies focused on weight‐ and length/height‐based metrics as proxies for excess adiposity, which are widely used given the low cost and ease of weight and height measurement, especially in younger children. Several cohorts also measured waist circumferences and skinfold thicknesses, which are highly correlated with fat mass in children.^69^ One cohort directly measured adiposity via air displacement plethysmograhy (the Healthy Start Study) and four cohorts assessed body composition via bioimpedance analysis (The Mount Sinai Children's Environmental Health Study, ELEMENT, CHAMACOS, and CCCEH).

Despite variability in the types of EDCs assessed, biospecimens used, and method of adiposity assessment, we noted a few consistent trends in the findings. With respect to EDC exposure during the prenatal and perinatal periods, exposure to POPs (e.g., PFAS, PBDE, DDT, and DDE) was associated with lower birthweight and lower % neonatal fat mass, even after accounting for known determinants of neonatal adiposity. Potential mechanisms include an effect of POPs on shorter gestation length (though many studies accounted for gestational age at delivery as a covariate) and physiological consequences of oxidative stress on fetal growth.^70^ On the other hand, in utero exposure to short‐lived chemicals—namely, DAPs and phthalates—was generally unrelated to birthweight, although there was suggestive evidence that certain phthalates (e.g., MCPP) were associated with higher birthweight in the presence of maternal hyperglycemia.^54^ Similarly, one study reported that in utero organophosphate pesticide exposure (via maternal urinary DAP concentrations) was related to higher birthweight, but only among infants of mothers who had a specific genotype,^35^ pointing toward relevant biological interactions to test in future studies.

When considering the associations of prenatal and perinatal EDC exposure on obesity beyond infancy, sex‐specific associations began to emerge. We noticed a consistent pattern that prenatal POPs exposure corresponded with higher adiposity in boys but not girls. Exposure to short‐lived chemicals, like phenols and phthalates, also was associated with adiposity in a sex‐specific fashion, although findings are inconsistent. The lack of consistency in findings could be attributed to the various ways in which in utero exposure was handled in the statistical analyses (e.g., average across pregnancy vs. trimester‐specific and assessment of individual chemicals vs. summary scores or latent variables), and the extent to which assessment of short‐lived chemicals in these studies is an accurate representation of true exposure.

Finally, when considering the relatively small body of literature on EDC exposure and obesity‐related outcomes during childhood and/or adolescence, we noted that despite differences in study design, chemicals of interest, and whether or not sex‐specific associations were considered, exposure to short‐lived chemicals used as plasticizers and in personal care products (i.e., BPA and phthalates) was associated with higher adiposity in girls and lower adiposity in boys. Such discrepancies between males and females are likely driven, in part, by differences in the endocrine regulation system and fat deposition in pre‐ and peripubertal youth.^71^, ^72^

Limitations of literature reviewed herein include the fact that only two Latin American countries were represented and that studies of mixed race/ethnic groups did not report race/ethnicity‐specific estimates (thereby hindering inference to Latino/Hispanic youth). Additionally, none of the studies distinguished between indigenous versus nonindigenous segments of Latino/Hispanic populations—a topic of relevance given that indigenous persons have distinct exposure profiles and obesity‐related disparities that warrant special attention.^17^ We also noted a general lack of biomonitoring of human exposure in Latino/Hispanic populations. Finally, because this review was narrative (as opposed to a systematic review) and focused on the most recent biomedical evidence (i.e., within the last decade) archived on PubMed/Medline, there may be other relevant studies that were not included.

### 4.2 Gaps in knowledge, future directions, and research priorities

#### 4.2.1 Gaps in knowledge

As noted earlier, the life stage during which EDC exposure has received the most attention is the prenatal and perinatal periods in relation to birthweight and gestation length. This focus is likely related to greater phenotypic plasticity during this timeframe (likely due to the lability of the fetal epigenome^73^), and thus, greater potential for behaviors and environmental factors to impact long‐term health.^74^, ^75^ While continued research on EDC exposure during the prenatal and perinatal periods will undoubtedly be beneficial, the prenatal period is but one sensitive period during early life (Figure 1). Therefore, studies that consider independent and joint effects of EDC exposure across multiple sensitive periods of development will aid in identifying the most relevant life stages within which interventions may have the largest impact on the reduction of obesity‐related disease. This is particularly important among youth of Latino/Hispanic origin given evidence of race/ethnic differences in timing and tempo of puberty.^76^ Moreover, we found that evidence surrounding EDC exposure during childhood and adolescence in relation to obesity‐related health was scant, and not all studies considered sex‐specific (in addition to race/ethnicity‐specific) associations, which are likely to emerge and diverge from childhood onward. A better understanding of specific culprits of excess adiposity and metabolic risk will unveil the most effective and timely actions for preventive efforts.

Many studies assessing exposure to EDCs during the prenatal and perinatal periods used a single maternal biospecimen to represent the entire pregnancy, thereby precluding the ability to disentangle trimester‐specific effects. Additionally, it is unclear whether EDC levels measured in maternal biospecimens reflect maternal exposure specifically during pregnancy or the woman's lifetime exposure. This issue is particularly relevant for long‐lived and lipophilic EDCs that may be retained in maternal tissues,^28^ which may result in continued fetal exposure in the absence of acute exposure. This concept has important implications for a potential biological interaction between maternal peripartum weight status and EDC exposure on offspring health, as well as for similar interactions between a child's own weight status and EDC exposure.

#### 4.2.2 The role of policy regulation

The United States and Canada are ecological examples of the effectiveness of policy regulations to reduce EDC exposure.^77^ In the United States, policies to reduce or prohibit use of EDCs in consumer products have reduced exposure. For example, DEHP, DBP and benzyl butyl phthalate use have been targets of regulation since 2008.^77^ Accordingly, data from the National Health and Nutrition Examination Survey (NHANES) showed that US concentrations of EDC metabolites in blood, serum, and urine decreased in the last decade.^78^ Despite implementation of such policies, US Latino/Hispanic populations still incur disproportionate exposures to air pollutants, pesticides, and toxic industrial chemicals.^79^, ^80^ Reasons for this disparity are complex.^81^ Additional surveillance and research is warranted to better understand sociocultural determinants of environmental health of Latino and Hispanic youth in the United States, and to develop the appropriate intervention efforts and initiatives.

Though it is challenging to make inference on the effectiveness of policy to regulate EDC exposure in Latin America due to a lack of surveillance in these settings (Table S2), cross‐country comparisons allow us to extrapolate. In the ELEMENT Project, Lewis et al. found that pregnant women in Mexico City have up to threefold higher concentrations of MBP (~65 ng/ml during the third trimester) and MEHP (~6.9 ng/ml during the third trimester) than adult women (MBP: 17.0–21.6 ng/ml, MEHP: 2.2–4.2 ng/ml) in US NHANES 1999–2004.^78^ Similar trends are observed for Mexican children and adolescents.^78^, ^82^ A recent study conducted among pregnant women in Mexico reported no declines in phthalate metabolite concentrations over time.^83^ In fact, urinary concentrations of diisobutyl phthalate (DiBP) and butylbenzyl phthalate (BBzP) metabolites were higher among women enrolled in a cohort study from 2008 to 2010 than to those enrolled in 2007. Similarly, concentrations of DBP, DiBP, and BBzP metabolites assessed among women enrolled from 2007 to 2011 were higher than those reported for a similar population of women studied from 1997–2005.^83^

Such findings point toward a need to monitor and regulate use of EDCs in foods,^84^, ^85^ food containers,^86^ personal care products, and common household items. In the Pan‐American Health Organization (PAHO)'s 5‐year Plan of Action to stem childhood obesity (2014–2019), current environmental conditions were identified as a cause of overweight and obesity, with dietary preferences and access, as well as trade and agriculture policies being the main determinants of the quality of food supplies. PAHO's recommendations include fiscal policies and other incentives for production and consumption of healthy foods, regulation of marketing of unhealthy foods, better labeling of processed food and drink products, and improvement of school food and increased physical activity among schoolchildren.^87^ We suggest similar recommendations and policies targeting the use of EDCs in food and food products, especially among pregnant women, infants, children, and adolescents. However, such policies will only have measurable impacts on obesity reduction if they are backed up by relevant actions, including population education, enforcement, and program implementation.

#### 4.2.3 Research priorities

Key research priorities include detailed biomonitoring of EDC exposure levels in Latin American countries and communities of predominantly Latino/Hispanic populations. Following implementation of policies to regulate use of EDCs in agriculture, personal care items, and household products (an effort for which assessment of feasibility is beyond the scope of this review), there will be need to assess the efficacy of such policies to reduce exposure to chemicals and any subsequent impacts on obesity‐related health.

## 5 CONCLUSIONS

The evidence reviewed herein implicates early life EDC exposure in the etiology of childhood obesity. Despite the relatively large body of literature on this topic, EDCs are not typically included as points of intervention in obesity prevention frameworks, possibly reflecting a need for additional research yielding consistent and reproducible findings across study populations, age groups, and settings. Regardless, pregnant women, infants, and young children are populations of special concern. Therefore, promoting education, awareness, and vigilance of EDC exposure in these populations is especially important when considering EDCs as an actionable target for childhood obesity prevention programs. Such efforts are especially salient for Latin American countries where industrialization is increasing EDC exposure, as well as in regions of the United States comprising large Latino/Hispanic populations and/or where agricultural work or other jobs with a high burden of chemical exposure (e.g., firefighting, textile, and paper manufacturing) represent a large proportion of the economic output.

Ultimately, policy regulations are needed to reduce the production and release of EDCs into the environment. Such systems‐level regulations may have wider ranging impacts than recommendations that put the onus on individuals to change behaviors and lifestyle, although in all likelihood both “top‐down” and “bottom‐up” activities will be needed to produce a measurable impact on obesity and other aspects of human health (e.g., smoking cessation and tobacco control in the United States resulting from top‐down efforts like policies to control smoking indoors and tobacco taxes, as well as bottom‐up efforts comprising disseminations of health information and education). Given that public policy draws insight from research findings, effective communication and engagement among scientists and stakeholders (business leaders, regulators, and politicians) will be instrumental to achieve the shared ultimate goal of improving health.

## CONFLICT OF INTEREST

The authors have no conflicts to disclose.

## Supporting information

**Table S1.** Key mechanisms through which endocrine disrupting chemicals may lead to excess weight gain and/or obesity

**Table S2.** Summary of policies regulating the use of endocrine disrupting chemicals in the United States, Canada, and selected Latin America countries*
