# Supplementary material for: Exposure to obesogenic endocrine disrupting chemicals and obesity among youth of Latino or Hispanic origin in the United States and Latin America: A lifecourse perspective
Source: Obes Rev. 2021 May 5;22(Suppl 3):e13245. doi: 10.1111/obr.13245 (PMC8217151; doi:10.1111/obr.13245)
Supplement: Supplementary file 1 — Table S1. Key mechanisms through which endocrine disrupting chemicals may lead to excess weight gain and/or obesity [file OBR-22-e13245-s002.docx]

| Supplemental Table 1 Key mechanisms through which endocrine disrupting chemicals may lead to excess weight gain and/or obesity | | | | | |
| --- | --- | --- | --- | --- | --- |
|  | **Activation of PPARs** | **Sex hormone biosynthesis/function** | **Thyroid function** | **Appetite & satiety** | **References** |
| *Persistent organic pollutants (POPs)* | | | | | |
| PFAS | ✓ |  |  |  | ^1–4^ |
| OCPs | ✓ | ✓ |  |  | ^5–7^ |
| PCBs | ✓ | ✓ | ✓ |  | ^7–9^ |
| PBDEs | ✓ | ✓ | ✓ |  | ^7,10–13^ |
| *Short-lived chemicals* | | | | | |
| OPs | ✓ |  |  |  | ^14^ |
| Phthalates | ✓ | ✓ | ✓ | ✓ | ^7,13,15–17^ |
| BPA | ✓ | ✓ | ✓ | ✓ | ^13,18–21^ |
| PPARs: peroxisome proliferator activated receptors; PFAS: per- and polyfluoroalkyl substances; OCPs: organochlorine pesticides; PCBs: polychlorinated biphenyls; PBDEs: polybrominated diphenyl ethers; OPs: organophosphorus pesticides; BPA: bisphenol A. | | | | | |

**References**

1. Ma Y, Yang J, Wan Y, et al. Low-level perfluorooctanoic acid enhances 3 T3-L1 preadipocyte differentiation via altering peroxisome proliferator activated receptor gamma expression and its promoter DNA methylation. *J Appl Toxicol*. 2018;38(3):398-407. doi:10.1002/jat.3549

2. Watkins AM, Wood CR, Lin MT, Abbott BD. The effects of perfluorinated chemicals on adipocyte differentiation in vitro. *Mol Cell Endocrinol*. 2015;400:90-101. doi:10.1016/j.mce.2014.10.020

3. Rosen MB, Lee JS, Ren H, et al. Toxicogenomic dissection of the perfluorooctanoic acid transcript profile in mouse liver: evidence for the involvement of nuclear receptors PPAR alpha and CAR. *Toxicol Sci*. 2008;103(1):46-56. doi:10.1093/toxsci/kfn025

4. Vanden Heuvel JP, Thompson JT, Frame SR, Gillies PJ. Differential activation of nuclear receptors by perfluorinated fatty acid analogs and natural fatty acids: a comparison of human, mouse, and rat peroxisome proliferator-activated receptor-alpha, -beta, and -gamma, liver X receptor-beta, and retinoid X rec. *Toxicol Sci*. 2006;92(2):476-489. doi:10.1093/toxsci/kfl014

5. Moreno-Aliaga MJ, Matsumura F. Effects of 1,1,1-trichloro-2,2-bis(p-chlorophenyl)-ethane (p,p’-DDT) on 3T3-L1 and 3T3-F442A adipocyte differentiation. *Biochem Pharmacol*. 2002;63(5):997-1007. doi:10.1016/s0006-2952(01)00933-9

6. Kim J, Sun Q, Yue Y, et al. 4,4’-Dichlorodiphenyltrichloroethane (DDT) and 4,4’-dichlorodiphenyldichloroethylene (DDE) promote adipogenesis in 3T3-L1 adipocyte cell culture. *Pestic Biochem Physiol*. 2016;131:40-45. doi:10.1016/j.pestbp.2016.01.005

7. Legler J, Hamers T, van Eck van der Sluijs-van de Bor M, et al. The OBELIX project: early life exposure to endocrine disruptors and obesity. *Am J Clin Nutr*. 2011;94(6 Suppl):1933S-1938S. doi:10.3945/ajcn.110.001669

8. Su S, Wu G, Cheng X, et al. Oleanolic acid attenuates PCBs-induced adiposity and insulin resistance via HNF1b-mediated regulation of redox and PPARgamma signaling. *Free Radic Biol Med*. 2018;124:122-134. doi:10.1016/j.freeradbiomed.2018.06.003

9. Arsenescu V, Arsenescu RI, King V, Swanson H, Cassis LA. Polychlorinated biphenyl-77 induces adipocyte differentiation and proinflammatory adipokines and promotes obesity and atherosclerosis. *Environ Health Perspect*. 2008;116(6):761-768. doi:10.1289/ehp.10554

10. Wen Q, Xie X, Zhao C, et al. The brominated flame retardant PBDE 99 promotes adipogenesis via regulating mitotic clonal expansion and PPARgamma expression. *Sci Total Environ*. 2019;670:67-77. doi:10.1016/j.scitotenv.2019.03.201

11. Tung EWY, Boudreau A, Wade MG, Atlas E. Induction of adipocyte differentiation by polybrominated diphenyl ethers (PBDEs) in 3T3-L1 cells. *PLoS One*. 2014;9(4):e94583. doi:10.1371/journal.pone.0094583

12. Talsness CE, Kuriyama SN, Sterner-Kock A, et al. In utero and lactational exposures to low doses of polybrominated diphenyl ether-47 alter the reproductive system and thyroid gland of female rat offspring. *Environ Health Perspect*. 2008;116(3):308-314. doi:10.1289/ehp.10536

13. Grun F, Blumberg B. Endocrine disrupters as obesogens. *Mol Cell Endocrinol*. 2009;304(1-2):19-29. doi:10.1016/j.mce.2009.02.018

14. Blanco J, Guardia-Escote L, Mulero M, et al. Obesogenic effects of chlorpyrifos and its metabolites during the differentiation of 3T3-L1 preadipocytes. *Food Chem Toxicol*. 2020;137:111171. doi:10.1016/j.fct.2020.111171

15. Janesick A, Blumberg B. Minireview: PPARgamma as the target of obesogens. *J Steroid Biochem Mol Biol*. 2011;127(1-2):4-8. doi:10.1016/j.jsbmb.2011.01.005

16. Feige JN, Gelman L, Rossi D, et al. The endocrine disruptor monoethyl-hexyl-phthalate is a selective peroxisome proliferator-activated receptor gamma modulator that promotes adipogenesis. *J Biol Chem*. 2007;282(26):19152-19166. doi:10.1074/jbc.M702724200

17. Hatch EE, Nelson JW, Stahlhut RW, Webster TF. Association of Endocrine Disruptors and Obesity: Perspectives from Epidemiologic Studies. *Int J Androl*. 2010;33(2):324-332.

18. Ariemma F, D’Esposito V, Liguoro D, et al. Low-Dose Bisphenol-A Impairs Adipogenesis and Generates Dysfunctional 3T3-L1 Adipocytes. *PLoS One*. 2016;11(3):e0150762. doi:10.1371/journal.pone.0150762

19. Phrakonkham P, Viengchareun S, Belloir C, Lombes M, Artur Y, Canivenc-Lavier M-C. Dietary xenoestrogens differentially impair 3T3-L1 preadipocyte differentiation and persistently affect leptin synthesis.*J Steroid Biochem Mol Biol*. 2008;110(1-2):95-103. doi:10.1016/j.jsbmb.2008.02.006

20. Masuno H, Kidani T, Sekiya K, et al. Bisphenol A in combination with insulin can accelerate the conversion of 3T3-L1 fibroblasts to adipocytes. *J Lipid Res*. 2002;43(5):676-684.

21. Casals-Casas C, Desvergne B. Endocrine disruptors: from endocrine to metabolic disruption. *Annu Rev Physiol*. 2011;73:135-162. doi:10.1146/annurev-physiol-012110-142200
