# Supplementary material for: Exposure to obesogenic endocrine disrupting chemicals and obesity among youth of Latino or Hispanic origin in the United States and Latin America: A lifecourse perspective
Source: Obes Rev. 2021 May 5;22(Suppl 3):e13245. doi: 10.1111/obr.13245 (PMC8217151; doi:10.1111/obr.13245)
Supplement: Supplementary file 2 — Table S2. Summary of policies regulating the use of endocrine disrupting chemicals in the United States, Canada, and selected Latin America countries* [file OBR-22-e13245-s001.docx]

**Supplemental Table 2** Summary of policies regulating the use of endocrine disrupting chemicals in the United States, Canada, and selected Latin America countries*

|  | **The United States and Canada** | **Latin America** |
| --- | --- | --- |
| ***Persistent organic pollutants (POPs)*** | | |
| Per- and polyfluoroalkyl substances (PFAS) | **United States:** No federal maximum contaminant level for PFAS exists; however, the United States (U.S.) Environmental Protection Agency (EPA) has established a lifetime health advisory level for p[erfluorooctanoic acid](http://www.ec.gc.ca/Toxiques-toxics/Default.asp?lang=en&n=F68CBFF1-1) (PFOA) and PFOS (p[erfluorooctane sulfonate](http://www.ec.gc.ca/Toxiques-toxics/Default.asp?lang=En&n=ECD5A576-1)) of 70 parts per trillion.  The “[PFAS Action Act of 2019](https://www.congress.gov/116/bills/hr535/BILLS-116hr535ih.xml)” was approved by the U.S. House Committee on Energy and Commerce in 2019. This bill contains 18 [chapters](https://rules.house.gov/sites/democrats.rules.house.gov/files/BILLS-116HR535-RCP116-45.pdf) that cover nearly every environmental statute and media that PFAS may impact, such as a drinking water regulation, a label for PFAS-free products, the establishment of a PFAS infrastructure grant program, a listing of PFAS substances as hazardous air pollutants, prohibition on unsafe waste incineration, guidance on minimizing the use of firefighting foam and other related equipment containing any PFAS.^1^  The Food and Drug Administration (FDA) is working with the EPA, the U.S.Department of Agriculture (USDA), the National Institutes of Health, the Centers for Disease Control and Prevention, and the Department of Defense, as well as with local, state governments and national organizations to assess theenvironmental contamination of PFAS in foods, and to review the authorized uses of PFAS in food contact applications.^2^  **Canada:** The import, manufacture, use, sale, and offer for sale of PFOS, PFOA, and long-chain p[erfluorocarboxylic acids](http://www.ec.gc.ca/Toxiques-toxics/Default.asp?lang=En&n=F68CBFF1-1) (LC-PFCAs), and products containing PFOS, PFOA, and LC-PFCAs, is prohibited (with a limited number of exemptions) in accordance with the [Prohibition of Certain Toxic Substances Regulations of 201](http://www.ec.gc.ca/lcpe-cepa/eng/regulations/detailReg.cfm?intReg=207)2.^3^ | In adherence to the commitments established in the Stockholm Convention (signed in 2001), several Latin American countries are working on plans to restrict or eliminate the use of some PFAS.  **Argentina:** As part of the Stockholm Convention, Argentina ratified its commitment to the elimination of PFOS. As of 2017, Argentina had not eliminated or restricted the use of PFOS or perfluorooctane sulfonyl fluoride (PFOSF).^4^  **Bolivia:** In 2004, Bolivia prepared a National Implementation Plan intending to present the strategies to achieve the goals concerning the elimination, substitution, and reduction of POPs. However, the plan does not detail the strategies for each of the pollutants.^5^  **Brazil:** In 2015, the country formulated an action plan to be implemented in the next few years to reduce and eliminate PFOS and PFOSF.^6^ They currently still in use.^7^  **Chile:** There is no evidence of the existence of regulations that aim to eliminate the production, use, import and export of PFOS.^8^  **Mexico:** Mexico is committed to eliminating the use of PFOS and PFOSF, with the exemptions and acceptable uses specified in the Stockholm Convention. As of 2016, the government was working on characterizing the use of PFOS substances and their applications, which were unknown.^9^ |
| Organochlorine pesticides (OCPs): Dichlorodiphenyltrichloroethane (DDT) and dichlorodiphenyldichloroethylene (DDE) | **United States:** The use of DDT was banned in 1972.^10^  **Canada:**  Most uses of DDT were discontinued in the 1970s. The sale or use of DDT constitutes a violation of the Pest Control Products Act.^11^ | In adherence to the Stockholm Convention, many Latin American countries prohibited the use of DDT.  **Argentina:** The use of DDT was prohibited in the 1990s.^12^  **Bolivia:** The use of DDT was prohibited in 2005.^13^  **Brazil:** DDT was banned from any use in the 90s, including agricultural, veterinary, and public health.^6^  **Chile:** Since 1984, there are regulations aimed to eliminate its production, use, import, and export. The Ministry of Agriculture - Agricultural and Livestock Service, prohibits its use in places and processes related to food development.^8^  **Mexico:** The use of DDT was banned in 1999.^14^ |
| Polychlorinated biphenyl (PCBs) | **United States:** PCBs were banned in 1979.^15^  **Canada:** In 1977, the import, manufacture, and sale of PCBs were made illegal. Release to the environment was made illegal in 1985. According to the legislation, the owners of PCB equipment (i.e., electrical equipment, hydraulic systems, heat exchangers, etc.) were allowed to continue using it until the end of its service life. Since 1988, the storage of PCBs has been regulated. Handling, transport, and destruction of PCB equipment are also regulated.^16^ | In adherence to the Stockholm Convention, many Latin American countries banned the production and import of equipment containing PCBs, and they established plans to correctly dispose of them after the end of their service life.  **Argentina**: The production, import, and commercialization of PCBs were prohibited in 2002. The use of PCBs containing products or equipment was also banned, and the industry had to replace them before 2010.^4^  **Bolivia:** The sale, concession, donation of dielectric oils, and equipment containing PBCs is prohibited. Activities that continue to use PCBs must take the appropriate measures (i.e., storage and collection) to prevent contamination by spills, leaks, or failures in the process.^5^  **Brazil:** In the 1970s, Brazil banned the use and sale of PCBs in new equipment. Over time, they also ceased the production and importing of PCB-containing equipment; however, they permitted existing equipment to remain in use until the end of their lifespan. The most recent efforts are centered on the management and phase-out of PCBs and PCB-containing equipment with a gradual and final elimination by 2028.^6^  **Chile:** PCBs use was prohibited in 2000, but they are still present in old industrial equipment (mining and electrical). Chile has regulations regarding the identification, management, and disposal of equipment and products containing or contaminated with PCBs. The plan is to have them eliminated by 2028.^17^  **Mexico:** PCBs are no longer produced but are still present in old electrical equipment. There are also present in contaminated solids, oils, and waste from the management of containers that had PCBs. There are norms that regulate the management and disposal of equipment containing PCBs and contaminated residues.^18^ |
| Polybrominated diphenyl ethers (PBDEs) | **United States:** Manufacture and import of c-pentaBDE and c-octaBDE were phased out in 2004. A phase-out of manufacture and import of c-decaBDE was started in 2010. The commitment was to end its production, import, and sales for all uses by the end of 2013.^19^  **Canada:** The import, manufacture, use, sale, and offer for sale of PBDEs, and products containing them was prohibited (with a limited number of exemptions) in accordance with the [Prohibition of Certain Toxic Substances Regulations, 201](http://www.ec.gc.ca/lcpe-cepa/eng/regulations/detailReg.cfm?intReg=207)2.^3^ | **Argentina:** the use of products containing penta-BDE and octa-BDE was discontinued in 2004. Deca-BDE was still in use in 2017. As of 2017, the country was working on regulations for the adequate disposal of products containing PBDEs.^4^  **Brazil:** Brazil does not have legislation that establishes control over industrial chemicals such as PBDEs. Public authorities do not have systematic information on the production, use, import and export of these substances.^6^  **Chile:** As of 2017, Chile was working on an updated national inventory of products containing PBDEs. No plan for the reduction or elimination of the use of PBDEs had been established.^8^  **Mexico:** Mexico stopped the production of penta-BDE and octa-BDE in 2004-2005. As of 2016, the country was working on regulations for the adequate disposal of products (i.e., car seats, carpets, electronics) containing these PBDEs at the end of their service life.^9^ |
| ***Short-lived chemicals*** | | |
| Organophosphates (OPs) | **United States:** Banned or severely restricted for residential uses on account of their effects on human health and the environment. The use of OPs for agricultural purposes, such as pesticides in fruits and vegetables, is still allowed, as is its use in the reduction of mosquitoes in public spaces (i.e., parks).^20^  Many states have monitoring exposure programs. As part of these programs, they measure cholinesterase activity in the blood of pesticide applicators. The FDA, USDA, EPA, and the Occupational Safety and Health Administration have established criteria on allowable levels of OPs in foods, the environment, and the workplace.^21^ EPA has placed special attention on chlorpyrifos exposure in children, given its health risks. The use of this pesticide indoors and outdoors is restricted.^22^  **Canada:** OPs are used extensively in agricultural and non-agricultural sites. Several of these pesticides have been voluntarily discontinued or have had their residential uses severely restricted. They are allowed but stringently regulated by Health Canada’s Pest Management Regulatory Agency.^23^ | **Argentina:** The production, import, fractionation, commercialization, and use of OPs active substances are prohibited in Argentina.^24^  **Bolivia:** Unregulated selling practices, bad storage habits, and the use of illegal pesticides in Bolivia are widespread.^25^  **Chile:** The use of OPs is allowed in Chile for agricultural purposes. The government has established regulations to monitor exposure in specific populations such as agricultural workers.^26,27^  **México:** The use of OPs is currently allowed in Mexico. OPs residues in livestock, dairy, and products are regulated through different Mexican Official Norms.^28^  **Brazil:** The use of OPs is currently allowed.^29^ |
| Phthalates | **United States:** Through the Consumer Product Safety Improvement Act of 2008, the U.S.Congress prohibited children’s toys or child care products containing concentrations of more than 0.1 percent of di-(2-ethylhexyl) phthalate (DEHP), dibutyl phthalate (DBP) or benzyl butyl phthalate (BBP). In 2017, the use of diisononyl phthalate (DINP), diisobutyl phthalate, di-n-pentyl phthalate (DPENP), di-n-hexyl phthalate, dicyclohexylphthalate in children’s toys and child care products inconcentrations above 0.1 percent were permanently prohibited.^30^  **Canada:** According to current regulations (**Canadian Phthalates Regulations, SOR/2016-188)**, the plastic or vinyl in any toy or child care product should not contain more than 1 000 mg/kg of DEHP, DBP, or BBP. The plastic or vinyl in any part of a toy or child care product that may be placed in the mouth of a child under 4 years of age should not contain more than 1 000 mg/kg of DINP, diisodecyl phthalate (DIDP) or di-n-octyl phthalate (DNOP).^31^ | **Argentina:** Phthalates are banned from use in childcare products that may be placed in the mouth of children under 3 years of age (i.e., pacifiers). They have also established regulations for the production, import, export, and commercialization of childcare products with phthalates: DBP, BBP, DEHP, DNOP, DINP, and DIDP.^12^  **Chile:** No regulations on the use of phthalates have been established.^32^  **Mexico:** Only the content of dimethyl phthalate and DBP in bug repellent and cosmetics (i.e., nail polish) are regulated. Regulations on the use of phthalates on other products, including childcare products, have not been established.^33,34^  **Brazil:** Some regulations are in place. Plastic or vinyl products should no contain >0.1% by mass of DEHP, DBP, and BBP. The plastic or vinyl of products intended for the use of children under 3 years of age should not contain >0.1% by mass of DEHP, DBP, BBP. DINP, DIDP, and DNOP.^35^  The use of some phthalates in personal care products, cosmetics, and perfumes, is regulated including DPENP, DBP, DEHP, BBP, and n-pentyl-isopentylphthalate.^36^ |
| Bisphenol A (BPA) | **United States:** The FDA ended its authorization of the use of BPA in[baby bottles](https://en.wikipedia.org/wiki/Baby_bottle) and infant formula packaging, based on market abandonment, not safety.^37^  **Canada:** The [Canada Consumer Product Safety Act](http://laws-lois.justice.gc.ca/eng/acts/C-1.68/index.html) of 2010 makes it illegal to manufacture, import, advertise or sell polycarbonate baby bottles with BPA.^38^ | **Argentina:** A ban on the production, import, and commercialization of baby bottles with BPA was established in 2012.^12^  **Brazil:** Since 2012, the use of BPA is prohibited in bottles and similar items intended for feeding infants.^39^  **Chile:** No regulations on the use of BPA have been established.^32^  **México:** An initiative was introduced and approved in the Senate in 2017 to regulate the use of plastics containing BPA. This initiative exhorted the Federal Commission for the Protection Against Sanitary Risks and the Ministry of Health to control the use of plastics containing BPA in foods and beverages and to raise awareness among the population on the health hazards of BPA. As of 2020, no regulations seem to be in place.^40^ |
| *This table shows policies and regulations in countries relevant to the studies included in the review.  **Abbreviations**  POPs: persistent organic pollutants; PFAS: per-and polyfluoroalkyl substances; U.S.: Unites States; EPA: Environmental Protection Agency; PFOA: p[erfluorooctanoic acid](http://www.ec.gc.ca/Toxiques-toxics/Default.asp?lang=en&n=F68CBFF1-1); PFOS: p[erfluorooctane sulfonate](http://www.ec.gc.ca/Toxiques-toxics/Default.asp?lang=En&n=ECD5A576-1); FDA: Food and Drug Administration; USDA: United States Department of Agriculture; LC-PFCAs: long-chain p[erfluorocarboxylic acids](http://www.ec.gc.ca/Toxiques-toxics/Default.asp?lang=En&n=F68CBFF1-1); PFOSF: perfluorooctane sulfonyl fluoride; DDT: dichlorodiphenyltrichloroethane; PCBs:polychlorinated biphenyls; PBDEs: polybrominated diphenyl ethers;OPs: organophosphorus pesticides; DEHP: di-(2-ethylhexyl) phthalate; DBP: dibutyl phthalate; BBP: benzyl butyl phthalate; DINP: diisononyl phthalate; DPENP: di-n-pentyl phthalate;DIDP: diisodecyl phthalate; DNOP: di-n-octyl phthalate; BPA: bisphenol A. | | |

**References**

1. Senate of the Unites States. H.R.535 - PFAS Action Act of 2019. https://www.congress.gov/bill/116th-congress/house-bill/535. Published 2019. Accessed February 24, 2020.

2. Food and Drug Administration. Per and Polyfluoroalkyl Substances (PFAS). https://www.fda.gov/food/chemicals/and-polyfluoroalkyl-substances-pfas. Published 2019. Accessed February 24, 2020.

3. Government of Canada. Regulations Amending the Prohibition of Certain Toxic Substances Regulations, 2012. https://pollution-waste.canada.ca/environmental-protection-registry/regulations/view?Id=131. Published 2016. Accessed February 21, 2020.

4. Ministerio de Ambiente y Desarrollo Sustentable. Actualización Del Plan Nacional de Aplicación Del Convenio de Estocolmo Sobre Los Contaminantes Orgánicos Persistentes (COPs) En La República Argentina. http://chm.pops.int/Portals/0/download.aspx?d=UNEP-POPS-NIP-Argentina-COP5.Spanish.pdf. Published 2017. Accessed February 24, 2020.

5. Galarza Coca M, Colomo C, Aguilar Alcalá P, López J. Plan Nacional de Implementación de La República de Bolivia Para El Cumplimiento Del Convenio de Estocolmo Sobre Contaminantes Orgánicos Persistentes. https://www.informea.org/en/plan-nacional-de-implementación-de-la-república-de-bolivia-para-el-cumplimiento-del-convenio-de. Published 2004. Accessed February 24, 2020.

6. Ministry of the Environment. National Implementation Plan Brazil: Stockholm Convention. http://chm.pops.int/Implementation/NationalImplementationPlans/NIPTransmission/tabid/253/ctl/Download/mid/13657/Default.aspx?id=23&ObjID=670. Published 2015. Accessed February 24, 2020.

7. Nascimento RA, Nunoo DBO, Bizkarguenaga E, et al. Sulfluramid use in Brazilian agriculture: A source of per- and polyfluoroalkyl substances (PFASs) to the environment. Environ Pollut. 2018;242 (Pt B):1436-1443.

8. Ministerio del Medio Ambiente. Plan Nacional De Implementación La Gestión de Los Contaminantes Orgánicos Persistentes En Chile 2018-2022.http://chm.pops.int/Portals/0/download.aspx?d=UNEP-POPS-NIP-Chile-COP7.Spanish.pdf.Published 2017. Accessed February 24, 2020.

9. Secretaría de Medio Ambiente y Recursos Naturales. Plan Nacional de Implementación México 2016. Convenio de Estocolmo Sobre Contaminantes Orgánicos Persistentes.http://chm.pops.int/Portals/0/download.aspx?d=UNEP-POPS-NIP-Mexico-COP5.Spanish.pdf.Published 2017. Accessed February 24, 2020.

10. Centers for Disease Control and Prevention. Dichlorodiphenyltrichloroethane (DDT). https://www.cdc.gov/biomonitoring/DDT_FactSheet.html. Published 2017. Accessed February 21, 2020.

11. Government of Canada. Toxic substances list: DDT. https://www.canada.ca/en/environment-climate-change/services/management-toxic-substances/list-canadian-environmental-protection-act/dichlorodiphenyltrichloroethane.html. Published 2012. Accessed February 21, 2020.

12. Departamento de Salud Ambiental. Químicos Prohibidos y Restringidos en Argentina. http://www.msal.gob.ar/images/stories/bes/graficos/0000000939cnt-quimicos_prohibidos_y_restringidos_2016.pdf. Published 2016. Accessed February 21, 2020.

13. Ministerio del Medio Ambiente y Agua. Lista de Plaguicidas Prohibidos y Restringidos En Bolivia. http://snia.mmaya.gob.bo/web/PDFs/Lista_Plaguicidas_Prohibidos_Restringidos.pdf. Published 2015. Accessed February 21, 2020.

14. Torres-Sanchez L, Lopez-Carrillo L. Human health effects and p,p’-DDE and p,p’-DDT exposure: the case of Mexico. Cien Saude Colet. 2007;12(1):51-60.

15. United States Environmental Protection Agency. Polychlorinated Biphenyls (PCBs). https://www.epa.gov/pcbs/learn-about-polychlorinated-biphenyls-pcbs#main-content. Published 2017. Accessed February 21, 2020.

16. Government of Canada. Toxic substances list: PCBs. https://www.canada.ca/en/environment-climate-change/services/management-toxic-substances/list-canadian-environmental-protection-act/polychlorinated-biphenyls.html. Published 2017. Accessed February 21, 2020.

17. Comision Nacional de Medio Ambiente. Plan Nacional de Implementación Para La Gestión de Los Contaminantes Orgánicos Persistentes (COPs) En Chile. Fase I: 2006-2010.http://chm.pops.int/Portals/0/download.aspx?d=UNEP-POPS-NIP-Chile-1.Spanish.pdf.Published 2005. Accessed February 21, 2020.

18. Secretaría de Gobernación. NORMA Oficial Mexicana NOM-133-SEMARNAT-2015, Protección ambiental-Bifenilos Policlorados (BPCs)-Especificaciones de manejo. http://www.dof.gob.mx/nota_detalle.php?codigo=5426547&fecha=23/02/2016. Published 2016. Accessed February 21, 2020.

19. United States Environmental Protection Agency. Assessing and Managing Chemicals under TSCA. https://www.epa.gov/assessing-and-managing-chemicals-under-tsca/polybrominated-diphenyl-ethers-pbdes#address. Published 2017. Accessed February 21, 2020.

20. Kozawa K, Aoyama Y, Mashimo S, Kimura H. Toxicity and actual regulation of organophosphate pesticides. Toxin Rev. 2009;28(4):245-254.

21. Centers for Disease Control and Prevention. Biomonitoring Summary Organophosphorus Insecticides: Dialkyl Phosphate Metabolites. https://www.cdc.gov/biomonitoring/OP-DPM_BiomonitoringSummary.html. Published 2017. Accessed February 24, 2020.

22. Jaga K, Dharmani C. Sources of exposure to and public health implications of organophosphate pesticides. Rev Panam Salud Publica/Pan Am J Public Heal. 2003;14(3):171-185.

23. Health Canada. Pesticides and Health. https://www.canada.ca/content/dam/hc-sc/migration/hc-sc/ewh-semt/alt_formats/hecs-sesc/pdf/pubs/contaminants/pesticides-eng.pdf. Published 2007. Accessed February 21, 2020.

24. Departamento de Salud Ambiental. Los Plaguicidas En La República de Argentina. http://www.msal.gob.ar/images/stories/bes/graficos/0000000341cnt-14-Plaguicidas_Argentina.pdf.Published 2014. Accessed February 21, 2020.

25. Haj-Younes J, Huici O, Jors E. Sale, storage and use of legal, illegal and obsolete pesticides in Bolivia. Cogent Food Agric. 2015;1:1008860

26. Servicio Agrícola y Ganadero. Plaguicidas y fertilizantes. http://www.sag.cl/ambitos-de-accion/plaguicidas-y-fertilizantes/78/registros. Published 2020. Accessed February 24, 2020.

27. Ministerio de Salud. Normas Sanitarias Para El Uso de Plaguicidas y Vigilancia de Trabajadores Expuestos. https://www.minsal.cl/wp-content/uploads/2015/11/Compendio-de-Normas-Sanitarias-para-Uso-y-Vigilancia-de-trabajadores-expuestos-a-Plaguicidas.pdf.Published 2014. Accessed February 24, 2020.

28. Ortíz I, Avila-Chávez MA, Torres LG. Plaguicidas en México: usos, riesgos y marco regulatorio. Rev Latinoam Biotecnol Ambient y Algal. 2014;4(1):26-46.

29. Dallegrave A, Pizzolato TM, Barreto F, Bica VC, Eljarrat E, Barceló D. Residue of insecticides in foodstuff and dietary exposure assessment of Brazilian citizens. Food Chem Toxicol an Int J Publ Br Ind Biol Res Assoc. 2018;115:329-335.

30. United States Consumer Product Safety Commission. Phthalates Business Guidance & Small Entity Compliance Guide. https://www.cpsc.gov/Business--Manufacturing/Business-Education/Business-Guidance/Phthalates-Information. Published 2019. Accessed February 24, 2020.

31. Minister of Justice. Phthalates Regulations. https://laws-lois.justice.gc.ca/PDF/SOR-2016-188.pdf. Published 2016. Accessed February 24, 2020.

32. Muñoz Llancao C, Parker Wichelhaus J. Disruptores endocrinos: información general, efectos en el organismo y su inclusión en contenedores plásticos reutilizables destinados al almacenaje de alimentos [undergraduate thesis]. Santiago, Chile 2017: Universidad Finis Terrae; 2007.

33. Bustamante Montes P. Mesa redonda VII. Necesidades regulatorias sobre los efectos de los plastificantes en la población infantil. Salud Publica Mex. 2007;49:72-75.

34. Secretaria de Gobernación. ACUERDO por el que se determinan las sustancias prohibidas y restringidas en la elaboración de productos de perfumería y belleza. http://dof.gob.mx/nota_detalle.php?codigo=5143790&fecha=21/05/2010. Published 2010. Accessed February 21, 2020.

35. INMETRO-Ministério do desenvolvimento indústria e comércio exterior. Portaria n.^o^ 369, de 27 de setembro de 2007. http://www.inmetro.gov.br/legislacao/rtac/pdf/RTAC001208.pdf. Published 2007. Accessed March 19, 2020.

36. Ministério da Saúde, Agência Nacional de Vigilância Sanitária. Resolução da diretoria colegiada – RDC n° 83, de 17 de Junho de 2016. Regulamento técnico Mercosul sobre lista de substâncias que não podem ser utilizadas em produtos de higiene pessoal, cosméticos e perfumes. http://bvsms.saude.gov.br/bvs/saudelegis/anvisa/2016/rdc0083_17_06_2016.pdf. Published 2016. Accessed May 19, 2020.

37. Food and Drug Administration. Bisphenol A (BPA): Use in Food Contact Application. https://www.fda.gov/food/food-additives-petitions/bisphenol-bpa-use-food-contact-application. Published 2018.Accessed February 24, 2020.

38. Minister of Justice. Canada Consumer Product Safety Act. https://laws-lois.justice.gc.ca/PDF/C-1.68.pdf. Published 2010. Accessed February 24, 2020.

39. Ministério da Saúde. Resolução Da Diretoria Colegiada – RDC N^o^ 56, de 16 de Novembro de 2012. http://portal.anvisa.gov.br/documents/10181/4048184/RDC_56_2012_.pdf/10f17ada-526e-434e-bc41-d0ec0b9e35cd?version=1.0. Published 2012. Accessed February 24, 2020.

40. Senado de la República. Gaceta del Senado. https://www.senado.gob.mx/64/gaceta_del_senado/documento/71742. Published 2017. Accessed February 25, 2020.
